# Increasing hydrophobicity in poly(amino acid) adjuvants elicits potent and durable anti-tumor immunity *via* coordinated cellular and humoral responses

**DOI:** 10.1016/j.bioactmat.2026.08.009

**Published:** 2026-08-19

**Authors:** Changjuan Qin, Hua Xin, Qi Wei, Qingyang Xiao, Guanqing Yang, Jianxun Ding

**Affiliations:** a Department of Thoracic Surgery, China-Japan Union Hospital of Jilin University, 126 Xiantai Street, Changchun, 130033, PR China; b State Key Laboratory of Polymer Science and Technology, Changchun Institute of Applied Chemistry, Chinese Academy of Science, 5625 Renmin Street, Changchun, 130022, PR China; c School of Applied Chemistry and Engineering, University of Science and Technology of China, 96 Jinzhai Road, Hefei, 230026, PR China

**Keywords:** Poly(amino acid) adjuvant, Hydrophobicity regulation, Cellular and humoral immunity, Tumor vaccine efficacy, Long-term immune memory

## Abstract

The coordinated activation of cellular and humoral immunity is essential for robust anti-tumor efficacy and durable immune memory in tumor vaccination. However, most current strategies are biased toward one immune arm, and the rational adjuvant design that efficiently engages both immune arms remains a challenge. Here, the hydrophobic segment content in an amphiphilic poly(amino acid) was optimized to boost both immune responses. The copolymers, poly(L-phenylalanine)_m_-*block*-poly(D-lysine)_10_ (m = 5, 10, or 15; F-_D_K), were synthesized with identical cationic components and different hydrophobic components. Increasing the phenylalanine ratio induced coordinated changes in nanoparticle size, surface charge, and structural rigidity, thereby enhancing dendritic cell uptake, TLR4-mediated activation, and antigen presentation. After subcutaneous injection into mice, F_15_-_D_K_10_/OVA, with longest chain of poly(L-phenylalanine), triggered robust CD8^+^ T cell cytotoxicity and strong B cell-mediated antibody response. Meanwhile, splenic central memory T cells and lymph node memory B cells increased by 1.46- and 2.19-fold, compared with the free ovalbumin (OVA). In the OVA-expressing Lewis lung cancer (LLC-OVA) therapeutic model, vaccines with increasing phenylalanine achieved tumor inhibition rates of 31.62%, 65.35%, and 97.81%. Importantly, F_15_-_D_K_10_/OVA achieved 100% tumor-free survival in the LLC-OVA prevention model. Even after two tumor re-challenges, 54.55% of mice remained tumor-free for 644 days, demonstrating durable antigen-specific protection in this model. A CT26 whole-cell-lysate study provided supporting evidence of therapeutic efficacy in one additional syngeneic tumor model. Within the tumor and antigen systems evaluated here, rational tuning of hydrophobic moieties in poly(amino acid) is an effective strategy to enhance tumor vaccine efficacy and long-term anti-tumor immune memory.

## Highlights

• Optimizing hydrophobic segments in poly(amino acid) enhances cellular and humoral immunity.
• Vaccination converts immunologically “cold” tumors into “hot” tumors and achieves effective tumor inhibition.
• Ultra-long antigen-specific immune memory is established against tumor rechallenge.

## Graphical abstract

**Figure:**
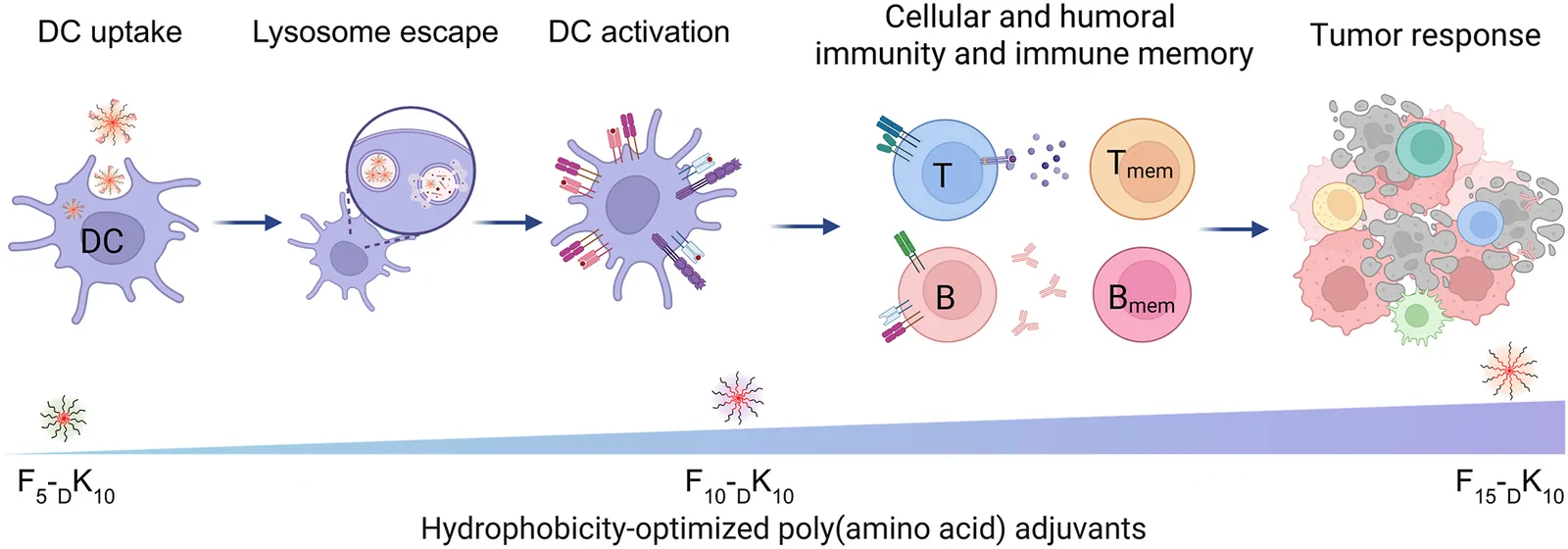

## 1 Introduction

Tumor vaccine is a central strategy in cancer immunotherapy. By delivering tumor-associated antigens, they aim to activate the immune system and elicit antigen-specific responses that enable precise recognition and elimination of malignant cells [1]. However, most current vaccines remain insufficient for effectively suppressing tumor progression and sustaining long-term immune responses [2,3]. From a mechanistic perspective, an ideal tumor vaccine should coordinate three interconnected axes. The first is the cellular immune axis, driven by CD8^+^ cytotoxic T lymphocytes (CTLs), which directly kill tumor cells [4,5]. The second is the humoral immune axis, which promotes B cell differentiation and the production of high-affinity, class-switched antibodies, thereby broadening antigen recognition and neutralization [6]. The third is the long-term immune memory axis, established by memory T cells and B cells, which underpins durable protection [7]. These axes are tightly coupled rather than independent, and their degree of coordination determines the magnitude, quality, and persistence of anti-tumor immunity. Achieving synchronized activation and efficiently converting short-term responses into durable immune memory remain central challenges in cancer vaccine development.

To address these limitations, recent efforts have increasingly focused on adjuvant development, due to their dual functions in enhancing antigen delivery and potentiating immune activation. Despite rapid advances in material design, current adjuvant strategies primarily optimize isolated aspects of the immune response rather than enabling coordinated and programmable immune regulation. As a result, these strategies struggle to simultaneously activate cellular and humoral immunity and establish durable immunological memory. For example, aluminum-based adjuvants, widely used in clinical settings, effectively induce humoral immunity but have limited capacity to stimulate cellular immunity, particularly CTL activation, and therefore fall short of the multidimensional requirements of anti-tumor responses. In contrast, emerging adjuvant platforms, including lipid nanoparticles, inorganic nanocarriers, and polymeric systems, primarily focus on optimizing physicochemical properties, such as particle size, surface charge, or hydrophilicity. These optimizations significantly enhance antigen delivery, lymph node targeting, and dendritic cell (DC) maturation, thereby strengthening T cell-mediated immunity. However, they often exhibit limited capacity to elicit robust humoral immunity, failing to generate high-affinity antibodies or establish long-lived B cell memory [[8], [9], [10], [11]]. Conversely, other design strategies instead prioritize humoral immunity, aiming to generate high-affinity, class-switched antibodies and long-lived plasma cells, but remain limited in their ability to induce robust cellular immunity [12,13]. Collectively, these limitations reflect a deeper conceptual challenge: The lack of a generalizable structure−immune response relationship that enables rational and predictive programming of immune outcomes across multiple arms of immunity. Therefore, developing adjuvants with intrinsic molecular programmability is becoming increasingly important for overcoming current bottlenecks in tumor vaccine design, particularly in enabling rational control over immune activation pathways. Modulation of hydrophobic−hydrophilic balance plays a central role in governing antigen delivery efficiency, cellular uptake pathways, and downstream immune activation [[14], [15], [16]]. Among emerging candidates, poly(amino acid)s have attracted significant attention due to their inherent biocompatibility, biodegradability, and tunable sequence structures. Their precise amino acid composition allows fine-tuning of the hydrophobic−hydrophilic balance at the molecular level. Nevertheless, a systematic understanding of how sequence-level programming is leveraged to coordinate cellular and humoral immune responses and drive durable immunological memory formation remains largely elusive.

**Scheme 1:**
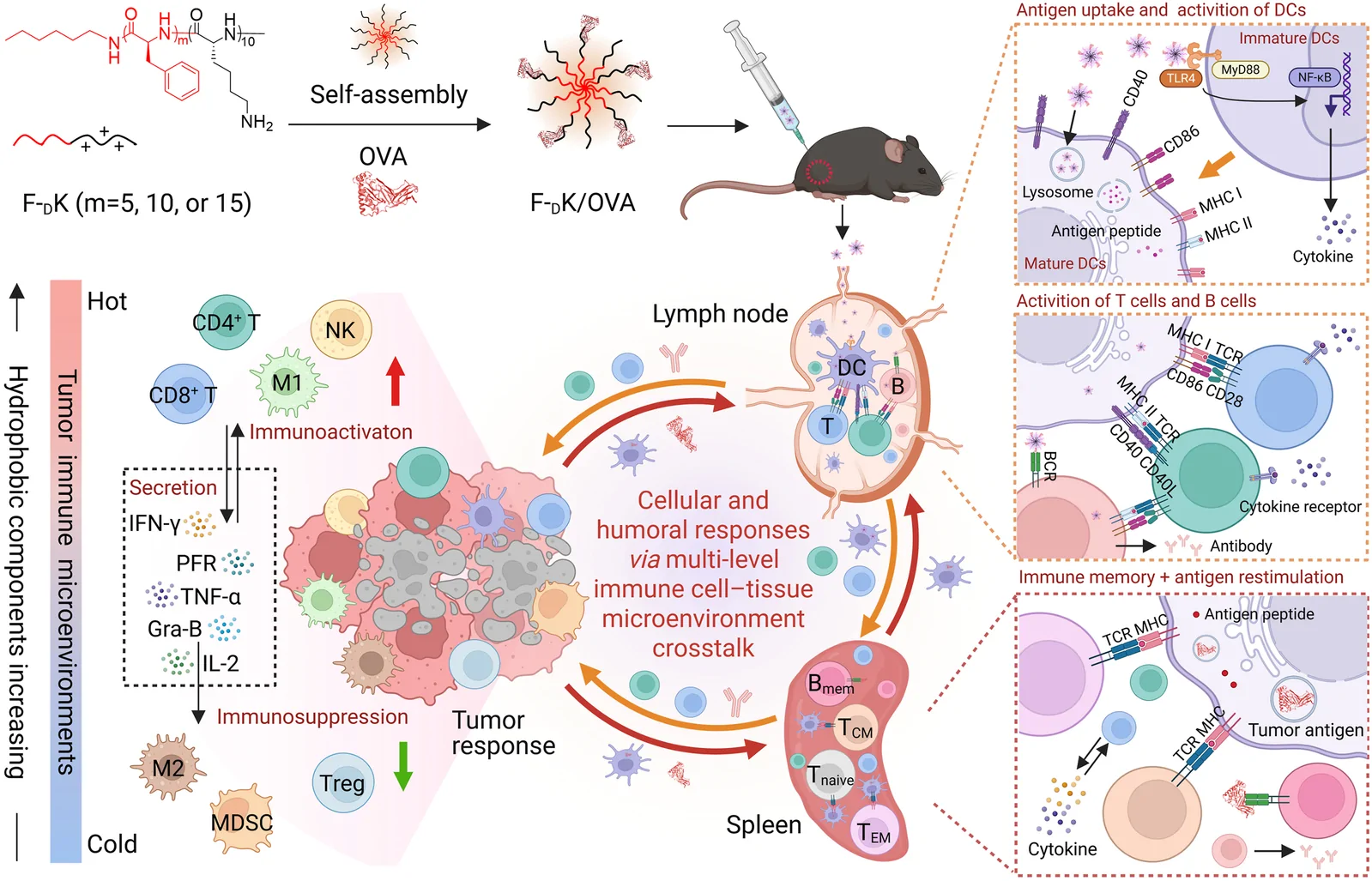
Mechanism of hydrophobic moieties-optimized F-_D_K/OVA as a tumor vaccine for cancer immunotherapy.

To address this question, this study developed a series of amphiphilic poly(amino acid) block copolymers, based on poly(L-phenylalanine)-*block*-poly(D-lysine) (F-_D_K). The length of hydrophobic L-phenylalanine (L-Phe) segment was varied to evaluate the immune response. The results showed that increasing hydrophobic moieties increases polymer particle size, zeta potential, and rigidity, thereby promoting cellular internalization [17]. By optimizing the hydrophobic ratio of poly(amino acid) adjuvants, these vaccines facilitated lysosomal escape, promoted TLR4-mediated activation, and enhanced antigen cross-presentation, collectively driving robust responses of T cells and B cells. Furthermore, longest poly(L-phenylalanine) vaccine, poly(L-phenylalanine)_15_-*block*-poly(D-lysine)_10_ (F_15_-_D_K_10_) with ovalbumin (OVA), promoted remodeled tumor immune microenvironments (TIMEs) in the OVA-expressing Lewis lung carcinoma (LLC-OVA) model. It increased populations of CD4^+^ and CD8^+^ T cells, natural killer (NK) cells, memory T cells, memory B cells, and anti-OVA antibody. Meanwhile, there were significant reductions in regulatory T cells (Tregs), tumor-associated macrophages (TAMs), and myeloid-derived suppressor cells (MDSCs). This coordinated multi-level crosstalk between immune cells and tissue microenvironments achieved potent anti-tumor effects and durable immune memory in the LLC-OVA model (Scheme 1). A therapeutic mouse colorectal carcinoma (CT26) model using whole-cell lysate as the antigen source was included as a second experimental system to further evaluate anti-tumor efficacy. By tailoring physicochemical properties with an optimized quantity of hydrophobic moieties, a cascade of favorable biological responses was elicited, generating a potent, amplified effect. This study provided experimental evidence and rational material strategies for next-generation amphiphilic poly(amino acid) vaccine adjuvants.

## 2 Materials and methods

### 2.1 Preparation of F-_D_K/OVA vaccines

Poly(L-phenylalanine)_m_-*block*-poly(D-lysine)_10_ (m = 5, 10, or 15; F_5_-_D_K_10_, F_10_-_D_K_10_, or F_15_-_D_K_10_) was synthesized by deprotecting poly(L-phenylalanine)_m_-*block*-poly(*N*(ε)-benzyloxycarbonyl-D-lysine)_10_ (F-Cbz-_D_K). The F-Cbz-_D_K precursor was synthesized through *n*-hexylamine (*n*-HA)-initiated ring-opening polymerization of L-phenylalanine *N*-carboxyanhydride (L-Phe NCA) and *N*(ε)-benzyloxycarbonyl-D-lysine *N*-carboxyanhydride (Cbz-D-Lys NCA) at defined monomer feed ratios. Detailed synthetic procedures are provided in the Supplementary data.

The F-_D_K/OVA vaccines were prepared *via* electrostatic adsorption. Briefly, F_5_-_D_K_10_, F_10_-_D_K_10_, and F_15_-_D_K_10_ were separately dissolved in 1× phosphate-buffered saline (PBS) to a final concentration of 1.0 mg mL^−1^ in 5.0-mL glass vials. The resulting solutions were sonicated for 90 min (4500 W, 18−28 °C) to induce nanoparticle formation. An OVA solution (0.2 mg mL^−1^) prepared in ultrapure water was subsequently added dropwise to each nanoparticle dispersion at room temperature (RT) under magnetic stirring for 15 min, yielding the F-_D_K/OVA vaccines.

### 2.2 Activation and cross-presentation assay of bone marrow-derived dendritic cells *in vitro*

Immature bone marrow-derived dendritic cells (BMDCs) were seeded into 12-well plates at a density of 1.0 × 10^6^ cells per well and treated with culture medium containing OVA (1.6 μg mL^−1^), F_5_-_D_K_10_/OVA (8.0 μg mL^−1^), F_10_-_D_K_10_/OVA (8.0 μg mL^−1^), F_15_-_D_K_10_/OVA (8.0 μg mL^−1^), or an equivalent volume of 1× PBS. After 12 h of incubation, BMDCs were harvested and stained with Zombie Aqua and fluorescently conjugated antibodies against CD11c, CD80, CD40, major histocompatibility complex class II (MHC II), and SIINFEKL peptide bound to the murine MHC I molecule H-2Kb (SIINFEKL−H-2Kb). Cells were analyzed by flow cytometry (FCM) using FlowJo v10.8.1. Culture supernatants collected from the corresponding wells were analyzed for interleukin-6 (IL-6), IL-12p70, interferon gamma (IFN-γ), and tumor necrosis factor alpha (TNF-α) using enzyme-linked immunosorbent assay (ELISA) kits according to the manufacturer's instructions.

### 2.3 Immune cell activation in lymph node

Female C57BL/6N mice were subcutaneously injected in the right dorsal flank with 100.0 μL of 1× PBS, OVA, F_5_-_D_K_10_/OVA, F_10_-_D_K_10_/OVA, or F_15_-_D_K_10_/OVA, with each formulation containing 20.0 μg of OVA per dose. On day 3, right inguinal draining lymph nodes (DLNs) were harvested, pooled in an equal volume of 1× PBS, and mechanically dissociated to obtain single-cell suspensions. Culture supernatants were collected and analyzed for IL-6, IL-12p70, IFN-γ, and TNF-α using ELISA kits according to the manufacturer's protocol. Cells were stained with Zombie Aqua and fluorophore-conjugated antibodies against CD11c, CD86, CD40, and MHC II, and subsequently analyzed by FCM using FlowJo v10.8.1. On day 5 after vaccination, the right inguinal DLNs were harvested and processed using the same procedure described above. Cells were then stained with Zombie Aqua and fluorophore-conjugated antibodies against CD11c, SIINFEKL−H-2Kb, CD3, CD4, CD8, IFN-γ, IL-4, CD19, CD86, and CD69, and analyzed by FCM.

### 2.4 Cancer therapy assay and biosafety assessment

On day 0, female C57BL/6N mice were subcutaneously injected in the right dorsal flank with 2.0 × 10^5^ LLC-OVA cells. After tumor inoculation, mice were randomly assigned to the experimental groups to ensure uniform baseline conditions. On days 4, 7, 11, 14, 18, 21, 25, and 28, mice received subcutaneous (*s.c.*) injections at the same site with 200.0 μL of 1× PBS, OVA, F_5_-_D_K_10_/OVA, F_10_-_D_K_10_/OVA, or F_15_-_D_K_10_/OVA, each containing 20.0 μg of OVA. Body weight, tumor length, and width were measured in a blinded manner to avoid subjective bias. These data were recorded at the indicated time points. Tumor volumes were calculated using Eq. (1), and tumor inhibition rates were subsequently determined using Eq. (2). Mice were humanely euthanized when tumor volume reached 2000 mm^3^, when severe weight loss (>20%) occurred, or when mice showed obvious mental depression and mobility impairment. No animals or data were excluded from this study, and all collected data were included in the final statistical analysis.

1 $$\begin{array}{c} \text{Tumor}\;\text{volume}\left({\text{mm}}^{3}\right)=\frac{\text{Length}\times {\text{Width}}^{2}}{2} \end{array}$$

2 $$\begin{array}{c} \text{Tumor}\;\text{inhibition}\;\text{rate}\;\left(\%\right)=\left(1-\frac{V_{\text{Treatment}}}{V_{\text{Control}}}\right)\times 100\% \end{array}$$

On day 31, the peripheral blood, tumors, DLNs, spleens, hearts, livers, lungs, and kidneys were harvested from treated mice. Tumor-derived single-cell suspensions were stained with Zombie Aqua and fluorophore-conjugated antibodies against CD45, CD3, CD8, CD4, forkhead box P3 (Foxp3), CD25, CD11b, F4/80, CD206, CD86, natural killer cell marker 1.1 (NK1.1), and granulocyte receptor 1 (Gr-1). Cells derived from lymph nodes were stained with Zombie Aqua and fluorophore-conjugated antibodies against CD45, CD3, CD8, and CD4. Splenocytes were stained with Zombie Aqua and fluorophore-conjugated antibodies against CD45, CD3, CD8, CD4, CD44, and CD62L. All samples were analyzed *via* FCM. ELISA kits were employed to quantify the concentrations of IL-2, IFN-γ, granzyme B (GzmB), perforin, and TNF-α within tumor homogenates. Whole blood samples were incubated at RT for 2 h, then centrifuged at 3500 rpm for 20 min to isolate serum. Serum concentrations of alanine aminotransferase (ALT), aspartate aminotransferase (AST), blood urea nitrogen (BUN), and creatinine (CRE) were determined using commercial assay kits. For histopathological evaluation, tumor tissues and major organs, including the heart, liver, spleen, lung, and kidney, were fixed in 4% (*W*/*V*) paraformaldehyde, embedded in paraffin, and stained with hematoxylin and eosin (H&E). Additional tumor sections were processed for immunohistochemical staining of CD8. All sections were imaged using a light microscope.

### 2.5 T cell memory and antigen-specific responses in the spleen

On days −21, −14, and −7, female C57BL/6N mice were subcutaneously injected in the right dorsal flank with 100.0 μL of 1× PBS, OVA, F_5_-_D_K_10_/OVA, F_10_-_D_K_10_/OVA, or F_15_-_D_K_10_/OVA, with each formulation containing 20.0 μg of OVA per dose. On day 0, the spleens were aseptically harvested and mechanically dissociated to generate single-cell suspensions. A portion of the splenocytes was immediately stained with Zombie Aqua and fluorophore-conjugated antibodies against CD3, CD4, CD8, CD44, and CD62L for FCM analysis. The remaining splenocytes were seeded into 48-well plates at 4.0 × 10^6^ cells per well and treated with a protein transport inhibitor. After 2 h of incubation, OVA peptide (257−264) (OVA_257−264_) was added at a concentration of 10.0 μg mL^−1^ and incubated for an additional 4 h. Cells were subsequently stained with the Zombie Aqua and fluorophore-conjugated antibodies against CD3, CD4, CD8, TNF-α, and IFN-γ according to standard protocols. Frequencies of OVA-specific CD8^+^ T cells were analyzed *via* FCM. In parallel experiments, splenocytes were cultured under identical conditions without protein transport inhibitors and stimulated with OVA_257−264_ for 48 h. Concentrations of TNF-α and IFN-γ in the culture supernatants were quantified using ELISA kits.

### 2.6 Antibody responses and immunomemory from B cells *in vivo*

Female C57BL/6N mice were subcutaneously injected in the right dorsal flank with 100.0 μL of 1× PBS, OVA, F_5_-_D_K_10_/OVA, F_10_-_D_K_10_/OVA, or F_15_-_D_K_10_/OVA, with each formulation containing 20.0 μg of OVA per dose. On day 7, blood was collected from the submandibular vein, yielding approximately 200.0 μL. Serum was obtained by centrifuging the blood at 3000.0 rpm for 20 min after standing for 2 h at RT. For antibody titer evaluation, the serum from different groups was diluted to 1/2^6^, 1/2^8^, 1/2^10^, 1/2^12^, 1/2^14^, 1/2^16^, 1/2^18^, and 1/2^20^. Then, the OD450 of the diluted serum was detected using an indirect ELISA for mouse IgG, IgG1, IgG2c, and IgM. Meanwhile, these mice were again injected in the right dorsal flank with 100.0 μL of 1× PBS, OVA, F_5_-_D_K_10_/OVA, F_10_-_D_K_10_/OVA, or F_15_-_D_K_10_/OVA, with each formulation containing 20.0 μg of OVA per dose. On day 14, the mice's blood was collected for an anti-OVA antibody titer test, as last time. Right DLNs and spleens were harvested, pooled in an equal volume of 1× PBS, and mechanically dissociated to obtain single-cell suspensions. Cells were stained with Zombie Aqua and fluorophore-conjugated antibodies against CD3, CD19, CD40, CD86, CD80, and CD138, and subsequently analyzed by FCM using FlowJo v10.8.1.

### 2.7 Cancer prevention assay

On days −21, −14, or −7, female C57BL/6N mice received a s.c. injection of 200.0 μL of 1× PBS, OVA, F_5_-_D_K_10_/OVA, F_10_-_D_K_10_/OVA, or F_15_-_D_K_10_/OVA, with each formulation containing 20.0 μg of OVA, into the left dorsal flank. On day 0, these mice were subcutaneously injected with 2.0 × 10^5^ LLC-OVA cells into the right dorsal flank. Tumor length and width and body weight were recorded at designated time points. Tumor volumes were calculated using Eq. (1), and vaccine protective efficacy was assessed using Eqs. (3) and (4). Mice were humanely euthanized when tumor volumes reached 2000 mm^3^.

3 $$\begin{array}{c} \text{Attack}\;\text{rate}\;\left(\%\right)=\frac{\text{Number}\;\text{of}\;\text{dead}\;\text{mice}}{\text{Total}\;\text{number}\;\text{of}\;\text{mice}}\times 100\% \end{array}$$

4 $$\begin{array}{c} \text{Vaccine}\;\text{protective}\;\text{efficacy}\;\left(\%\right)=\left(1-\frac{R_{\text{Treatment}}}{R_{\text{Control}}}\right)\times 100\% \end{array}$$

Tumor-free mice were re-challenged with tumors, while age-matched untreated mice served as controls. On day 121, 2.0 × 10^5^ LLC-OVA cells were subcutaneously injected into the right dorsal flank, and tumor volume, body weight, and survival were recorded. On day 287, the same number of LLC-OVA cells was subcutaneously injected into the left dorsal flank of tumor-naive mice, and the same parameters were recorded.

## 3 Results and discussion

### 3.1 Synthesis and characterizations of F-_D_K/OVA vaccines

**Fig. 1:**
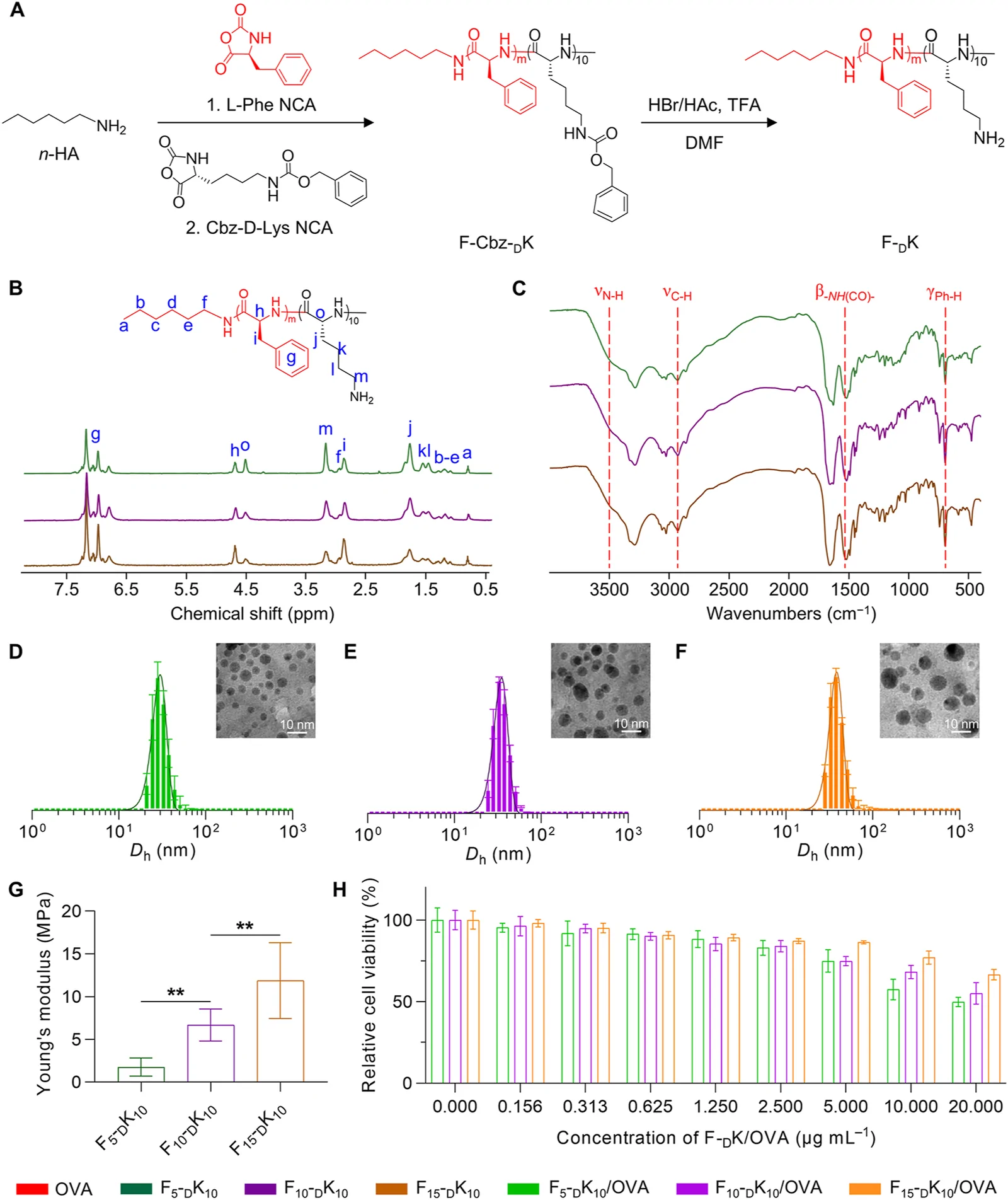
Synthesis and characterizations of F-_D_K/OVA. (A−C) Synthetic pathways (A), ^1^H NMR spectra in TFA-*d* (B), and FT-IR spectra of F-_D_K (C). (D−F) Hydrodynamic diameter (*D*_h_) measurements and TEM micrographs of F-_D_K/OVA nanoparticles. (G) Young's modulus of F-_D_K. (H) Relative viability of DC2.4 cells at 12 h post-treatment with varying concentrations of F-_D_K/OVA. All statistical data are represented as mean ± standard deviation (SD; *n* = 3 for D−G, *n* = 6 for H; NS, not significant; *P < 0.05, **P < 0.01, ***P < 0.001, ****P < 0.0001).

To optimize the hydrophilic−hydrophobic balance of cationic amphiphilic poly(amino acid) adjuvants for efficient immune activation, the ratio of hydrophobic L-Phe to hydrophilic D-Lys was systematically tuned. This strategy generated a series of poly(amino acid) materials with well-defined hydrophobicity gradients and precisely controlled architectures, namely F_5_-Cbz-_D_K_10_, F_10_-Cbz-_D_K_10_, and F_15_-Cbz-_D_K_10_ (Fig. 1A). Amino acids, as essential nutrients for the body, enhance the functions of immune cells [18]. Lys provides cationic charges for antigen binding and facilitates lysosomal escape. D-Lys was selected based on previous reports demonstrating stronger immune activation than that induced by L-Lys [19,20]. Phe contributes to hydrophobic interactions to enhance nanoparticle stability. However, further increasing the hydrophobic ratio would markedly reduce aqueous solubility and colloidal dispersibility, thereby compromising formulation feasibility, systemic circulation, and antigen delivery. Accordingly, the superior immunostimulatory performance of F_15_-_D_K_10_ identified in this work should be interpreted as the optimal formulation within the structural range investigated in the present study.

Proton (^1^H) and carbon-13 (^13^C) nuclear magnetic resonance (NMR) spectroscopy confirmed the chemical structures of two amino acid NCAs (Fig. S1 and S2, Supplementary data). Structural identification of F-Cbz-_D_K was further verified *via* ^1^H NMR spectroscopy and Fourier transform-infrared spectroscopy (FT-IR) (Fig. S3 and S4, Supplementary data). Gel permeation chromatography (GPC) results verified the molecular weights and dispersity of F_5_-Cbz-_D_K_10_, F_10_-Cbz-_D_K_10_, and F_15_-Cbz-_D_K_10_, confirming that F-Cbz-_D_K were synthesized with controllable polymerization and exhibited uniform chain-length distributions (Table S2, Supplementary data).

As shown in Supplementary Fig. S3, the resonance at 0.95 ppm (a) corresponds to the terminal methyl protons of initiator *n*-HA [21]. Signals at 4.75 ppm (h) and 4.55 ppm (o) were assigned to the α-carbon protons of Phe and Lys, respectively, within the poly(amino acid) backbone. The multiplet observed between 5.00 and 5.50 ppm (n) was attributed to the benzylic methylene protons of the Lys protecting group. Signals in the range of 6.98−7.50 ppm (g) were assigned to protons on the benzene ring. Using the signal area of peak (a) as a reference and setting it to three, the degrees of polymerization (DPs) of Phe and Lys were calculated from the signal area ratios of peaks (g), (h), (n), and (o). After deprotection of F-Cbz-_D_K with trifluoroacetic acid (TFA) and hydrogen bromide (33 wt% solution in acetic acid, HBr/HAc), the disappearance of signal (n) together with the reduced signal area of peak (g) confirmed successful removal of the protecting group. The same integration method was applied to determine the DPs in F-_D_K, confirming the successful synthesis of F_5_-_D_K_10_, F_10_-_D_K_10_, and F_15_-_D_K_10_ (Fig. 1B). FT-IR spectra (Fig. 1C and Fig. S4, Supplementary data) exhibited a characteristic signal at 1535 cm^−1^ corresponding to the amide II N−H in-plane bending vibration of the −NH(CO)− group. A signal at 1700 cm^−1^ was assigned to the carbonyl stretching vibration of benzyloxycarbonyl protecting group. Aliphatic C−H stretching vibrations appeared at 2935 cm^−1^, aromatic C−H out-of-plane bending vibrations were observed at 695 cm^−1^, and primary amine N−H stretching vibrations were detected at 3500 cm^−1^. Following deprotection, the band at 1700 cm^−1^ disappeared, and the band at 3500 cm^−1^ emerged, whereas the peak at 1535 cm^−1^ remained unchanged. These results indicate that the amide bonds within the polymer backbone remained intact and that the lysine protecting group was successfully removed.

The amphiphilic poly(amino acid) materials F_5_-_D_K_10_, F_10_-_D_K_10_, and F_15_-_D_K_10_ self-assembled into monodisperse nanoparticles in PBS at a concentration of 250.0 μg mL^−1^ *via* ultrasonication. Nanoparticles were measured by dynamic light scattering (DLS) at 25 °C with three independent replicates, and particle size distribution data were recorded in number mode. The diameters of 24.66 ± 4.11 nm (PDI: 0.278), 30.05 ± 4.89 nm (PDI: 0.309), and 32.01 ± 5.88 nm (PDI: 0.443) are for F_5_-_D_K_10_, F_10_-_D_K_10_, and F_15_-_D_K_10_, respectively. DLS and transmission electron microscopy (TEM) images confirmed uniform spherical morphology (Fig. S5, Supplementary data). The negatively charged model antigen OVA was efficiently loaded onto F-_D_K nanoparticles *via* electrostatic interactions with the protonated ε-amino group of lysine at physiological pH, forming an integrated tumor vaccine. The loading efficiency of OVA approached 100% at a nanoparticle-to-OVA mass ratio of 10:2 (Fig. S6, Supplementary data), confirming that F-_D_K nanoparticles function as an effective vehicle for antigen delivery. DLS with the same testing parameters and TEM analysis revealed that F-_D_K/OVA maintained uniform spherical morphology with slightly increased particle sizes compared with those of F-_D_K alone, measuring 29.96 ± 5.52 nm (PDI: 0.493), 34.99 ± 6.50 nm (PDI: 0.356), and 38.22 ± 6.78 nm (PDI: 0.331) for F_5_-_D_K_10_/OVA, F_10_-_D_K_10_/OVA, and F_15_-_D_K_10_/OVA, respectively (Fig. 1D−F). Furthermore, the nanoparticle stability of F-_D_K/OVA was tested. As shown in Supplementary Fig. S7, F-_D_K/OVA nanoparticles exhibited good stability under acidic conditions (pH 5.5) within 24 h, with only a slight and non-significant increase in size. It indicated their structural integrity upon lysosomal encounter. In contrast, incubation in 50% (*V*/*V*) fetal bovine serum (FBS), which mimics systemic circulation, led to noticeable size enlargement to approximately 200.0 nm at 24 h, particularly in F_15_-_D_K_10_/OVA. It was due to the protein corona effect, commonly observed with cationic nanomaterials [22,23]. Importantly, the nanoparticles are administered *via* *s.c.* injection, not intravenously. The protein content in lymph fluid is much lower than that in the blood [24,25]. The mild aggregation observed under high-protein conditions for a long time is unlikely to occur during lymphatic transit *in vivo*, as the nanoparticles (≤200.0 nm) are efficiently drained into lymph nodes before prolonged exposure to a protein-rich environment [26]. Therefore, our system is considered safe and stable for *s.c.* immunotherapy, where controlled particle size and colloidal behavior support effective lymphatic trafficking and immune activation.

Zeta potential measurements demonstrated that OVA exhibited a negative surface charge, whereas F-_D_K nanoparticles were positively charged (Fig. S8, Supplementary data). The zeta potential of F-_D_K slightly increased with increasing hydrophobic content. Given that the total number of amino group per monomer is constant, the observed rise in zeta potential suggests a higher surface charge density. This is reasonably attributed to an increased monomer aggregation number per nanoparticle, driven by prolonged hydrophobic domains. The slight increase in hydrodynamic diameter of the corresponding nanoparticles further supports this interpretation. A similar trend has been validated in recent studies. For instance, Qiao et al. demonstrated that, for polycationic delivery systems with identical total amino group, appropriately enhanced hydrophobicity effectively regulates nanoparticle self-assembly, ultimately leading to increased surface zeta potential [27]. Upon OVA loading, the zeta potential of F-_D_K/OVA decreased significantly, further confirming electrostatic adsorption and partial neutralization of surface amino group on F-_D_K nanoparticles. Atomic force microscopy (AFM) measurements revealed an increase in Young's modulus with increasing hydrophobicity (Fig. 1G), indicating enhanced material rigidity. These particle size distributions, surface charge characteristics, and rigidity profiles are favorable for lymphatic drainage and immune cell uptake [[28], [29], [30]].

The F-_D_K/OVA formulations are therefore expected to perform effectively in subsequent cellular and animal experiments. Given that cationic materials disrupt cell membranes and induce cell death at high concentrations [31], cytotoxicity assays were performed for F-_D_K and F-_D_K/OVA (Fig. 1H and Fig. S9A, Supplementary data). All three materials exhibited comparable cytotoxicity profiles, and a concentration below the half-maximal inhibitory concentration (IC_50_) was selected for subsequent cellular experiments (Fig. S9B, Supplementary data).

### 3.2 Uptake and lysosomal escape of F-_D_K/OVA in dendritic cells

**Fig. 2:**
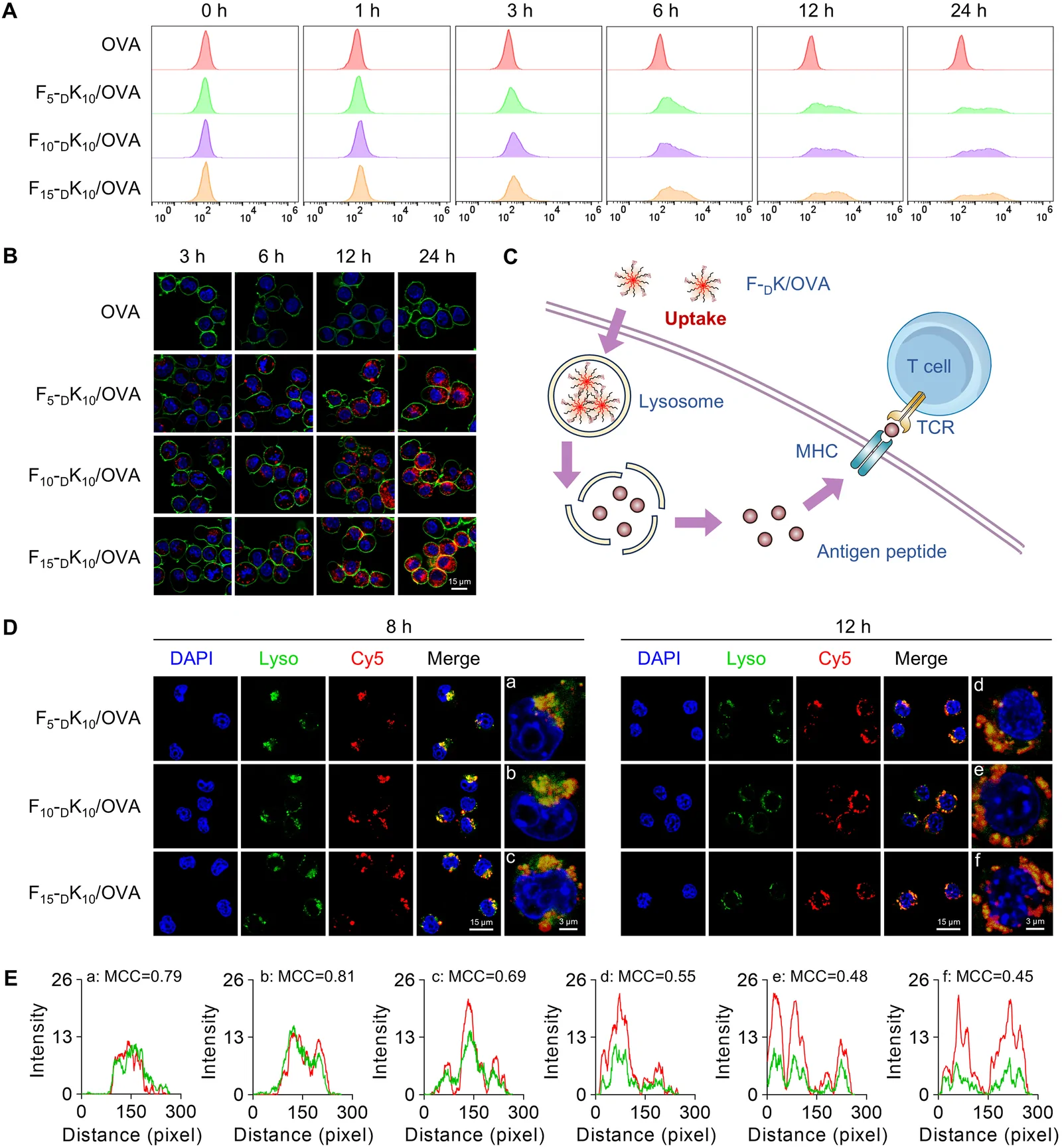
Effects of F-_D_K/OVA on uptake and lysosomal escape in DCs. (A) FCM analysis of DC2.4 cell endocytosis following treatment with FITC-labeled vaccines at 0, 1, 3, 6, 12, and 24 h. (B) CLSM images showing cellular uptake of Cy5-labeled vaccines in DC2.4 cells at 3, 6, 12, and 24 h. Blue, cell nucleus; Green, lysosome; Red, F-_D_K/OVA-Cy5. (C) Experimental strategy for antigen uptake, lysosomal escape, and cross-presentation in DCs of F-_D_K/OVA. (D) CLSM images illustrating lysosomal escape of Cy5-labeled vaccines in DC2.4 cells at 8 and 12 h. Blue, cell nucleus; Green, lysosome; Red, F-_D_K/OVA-Cy5. (E) Colocalization analysis of lysosome escape in DC2.4 cells of Fig. 2Da−f. Green, lysosome; red, F-_D_K/OVA-Cy5. MCC: Manders' Colocalization Coefficients. All CLSM images were captured with identical laser power, gain, exposure, and offset parameters for unbiased comparison.

Efficient antigen uptake is essential for DCs to initiate antigen processing and presentation, thereby determining the magnitude of downstream immune responses. To evaluate whether F-_D_K nanoparticles enhance antigen internalization, OVA was labeled with fluorescein isothiocyanate (FITC) and assembled with F-_D_K to generate F-_D_K/OVA−FITC complexes for real-time intracellular tracking. DC2.4 cells were incubated with F-_D_K/OVA−FITC for various durations, and intracellular FITC fluorescence intensity was quantified by FCM (Fig. S10A and S10B, Supplementary data). Compared with free OVA−FITC, all F-_D_K/OVA−FITC complexes exhibited significantly enhanced and time-dependent uptake (Fig. 2A and Fig. S10C, Supplementary data). Among these formulations, the highest Phe introduction ratios, F_15_-_D_K_10_/OVA−FITC, showed the highest cellular accumulation, with a 12.23-fold increase in fluorescence intensity compared with free OVA−FITC at 24 h. This enhanced uptake is attributed to the synergistic effects of cation-mediated electrostatic interactions and increased hydrophobic moieties. The positively charged nanoparticles promote association with negatively charged plasma membranes, while increased hydrophobic components may enhance interactions with hydrophobic membrane regions, thereby facilitating nanoparticle internalization [27,30,32,33]. In addition, a higher Phe content in F_15_-_D_K_10_ introduced abundant rigid aromatic rings, which may further promote cellular internalization [[34], [35], [36]]. Confocal laser scanning microscopy (CLSM) imaging further confirmed efficient cytosolic delivery of F-_D_K/OVA, with fluorescence signals occupying nearly the entire intracellular space after 24 h of incubation (Fig. 2B and Fig. S10D, Supplementary data).

Antigen uptake by DCs represents only the initial step in tumor vaccine activity. For effective immune activation, OVA must be transported to the lysosomes, processed into short peptides, escape into the cytosol, and subsequently be presented on MHC molecules for recognition and activation of T cells (Fig. 2C) [37,38]. To examine intracellular distribution, DC2.4 cells were treated with F-_D_K/OVA-cyanine 5 (Cy5), and lysosomes were stained for CLSM analysis (Fig. 2D). At 8 h, pronounced colocalization between red fluorescence signals from F-_D_K/OVA−Cy5 and green lysosome signals was observed, with colocalization increasing as vaccine hydrophobicity increased. These results suggest that vaccines with higher hydrophobicity are internalized more rapidly, generating a larger pool of early endosomes and providing kinetic and concentration advantages for subsequent lysosomal processing. At 12 h, reduced colocalization and attenuated lysosomal signals demonstrated lysosomal swelling and rupture, which facilitated the release of processed OVA peptides into the cytosol (Fig. 2E and Fig. S10E, Supplementary data) [39]. The lysosomal escape of F-_D_K/OVA block copolymers is primarily attributed to cationic-mediated lysosomal membrane disruption, where the local high concentration of Lys interacts electrostatically with negatively charged phospholipids of the lysosomal membrane, leading to content release into the cytosol [40]. Importantly, as shown in the preceding results, longer hydrophobic Phe segment increased nanoparticle size and zeta potential. It reflects a higher monomer packing number per nanoparticle. This yields a higher density of lysine amino group, enhancing lysosomal escape efficiency, which explains why F_15_-_D_K_10_/OVA escapes fastest. Efficient lysosomal escape ensures complete antigen processing and effective presentation, thereby enabling downstream T cell activation.

Collectively, these data demonstrate that F-_D_K with an optimized quantity of hydrophobic moieties enhances DC antigen uptake, promotes timely lysosomal trafficking, and facilitates efficient lysosomal escape, thereby supporting robust antigen processing and potent T cell activation.

### 3.3 Activation and antigen cross-presentation of bone marrow-derived dendritic cells by F-_D_K/OVA

**Fig. 3:**
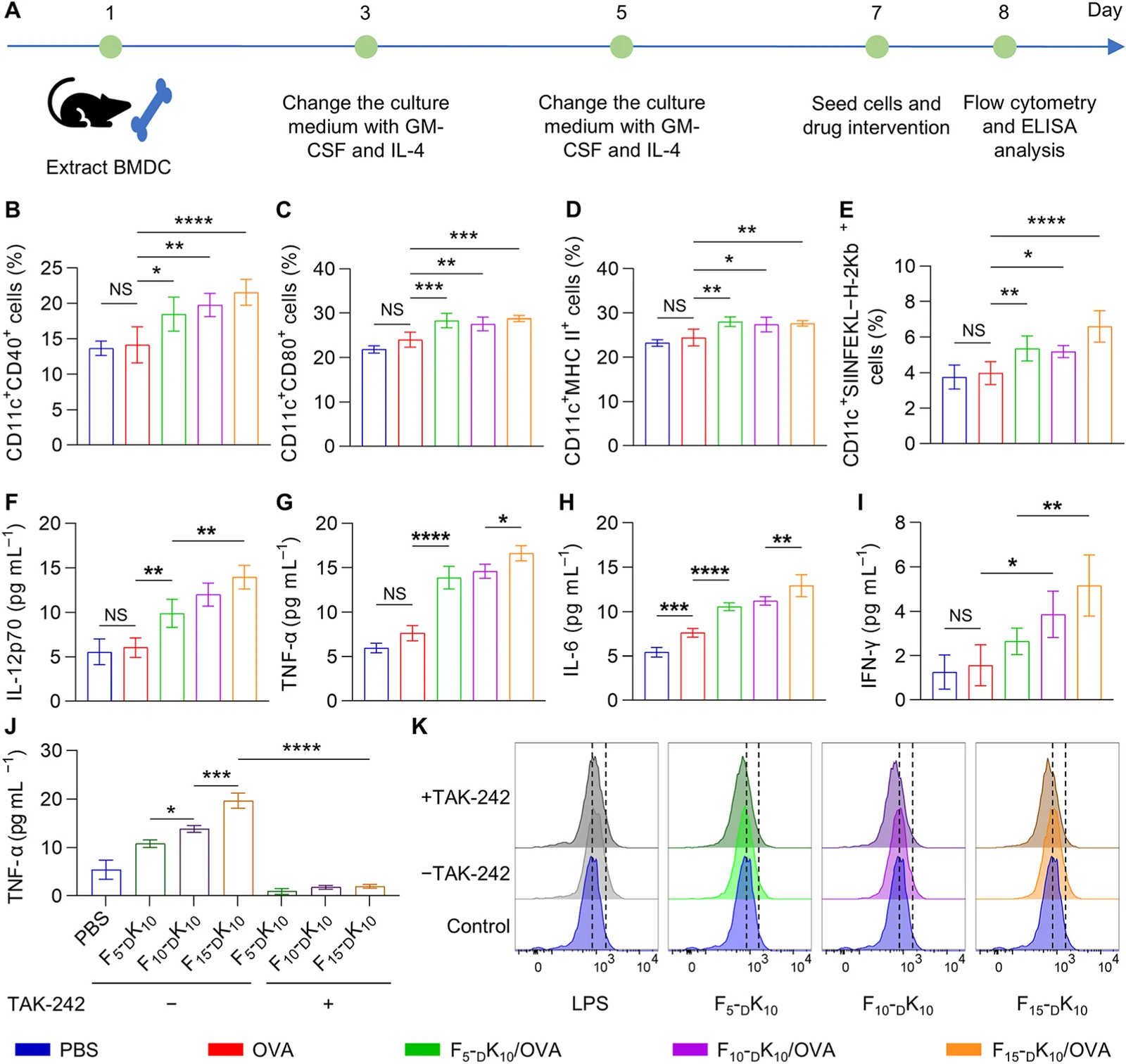
Effects and pathway of F-_D_K/OVA on activation and antigen cross-presentation in DCs. (A) Experimental strategy for BMDC activation and antigen cross-presentation induced *via* F-_D_K/OVA. (B−E) CD11c^+^CD40^+^ cells (B), CD11c^+^CD80^+^ cells (C), CD11c^+^MHC II^+^ cells (D), and CD11c^+^SIINFEKL−H-2Kb^+^ cells on BMDCs (E) following incubation with different treatments for 12 h. (F−I) IL-12 p70 (F), TNF-α (G), IL-6 (H), and IFN-γ concentrations in culture supernatants of BMDCs (I) following varying treatments for 12 h. (J,K) Concentrations of TNF-α in supernatant (J) and CD11c^+^CD80^+^ FCM analysis of DC2.4 cells (K) pretreated with TAK-242 (5.0 μM) or without TAK-242 for 2 h and then incubated with PBS, LPS, or F-_D_K for 12 h. All statistical data are represented as mean ± SD (*n* = 5 for B−I, *n* = 3 for J and K; NS, not significant; *P < 0.05, **P < 0.01, ***P < 0.001, ****P < 0.0001).

Given that F-_D_K/OVA markedly enhances antigen uptake and lysosomal escape in DCs, the capacity of these vaccines to activate DCs was further evaluated (Fig. 3A). Immature BMDCs were treated with F-_D_K/OVA for 12 h, and expression levels of activation markers CD40, CD80, and MHC II were quantified *via* FCM (Fig. 3B−D). Detailed gating strategies for FCM are provided in Supplementary Fig. S11A. Compared to OVA alone, all F-_D_K/OVA formulations significantly enhanced BMDC activation. The percentage of CD11c^+^CD40^+^ cells in the F_5_-_D_K_10_/OVA, F_10_-_D_K_10_/OVA, and F_15_-_D_K_10_/OVA groups increased in a hydrophobicity-dependent manner, reaching 1.29-, 1.45-, and 1.58-fold of the OVA group, respectively (Fig. 3B and Fig. S11B, Supplementary data). Antigen cross-presentation was evaluated by measuring surface expression of the SIINFEKL−H-2Kb complex. Compared with free OVA, F_5_-_D_K_10_/OVA, F_10_-_D_K_10_/OVA, and F_15_-_D_K_10_/OVA increased SIINFEKL−H-2Kb expression by 34.76%, 30.33%, and 66.17%, respectively (Fig. 3E). Consistent with these findings, ELISA analysis of culture supernatants revealed substantial increases in pro-inflammatory cytokines, including IL-12p70, TNF-α, IL-6, and IFN-γ (Fig. 3F−I). In particular, the F_15_-_D_K_10_/OVA formulation increased cytokine levels by 131.73%, 117.80%, 69.84%, and 230.05%, respectively, relative to the OVA group, in agreement with the FCM results. These cytokines promote T cell activation and amplify adaptive immune responses. Collectively, these data indicate that F-_D_K/OVA effectively activates BMDCs, enhances antigen cross-presentation, and elicits strong antigen-specific T cell responses, with immunostimulatory potency increasing with the number of hydrophobic moieties.

To elucidate the underlying activation mechanism, DCs were treated with the TLR4-specific inhibitor TAK-242. In the absence of inhibitors, DCs were activated. The levels of TNF-α in the F_5_-_D_K_10_, F_10_-_D_K_10_, and F_15_-_D_K_10_ groups were 2.00-, 2.56-, and 3.65-fold those of the PBS group, respectively. Conversely, upon inhibitor treatment, TNF-α secretion in the F-_D_K-treated groups returned to levels comparable to those in the PBS control (Fig. 3J). Concurrently, FCM analysis revealed that the proportion of mature DCs (CD11c^+^CD80^+^) was also completely abrogated following inhibitor administration (Fig. 3K and Supplementary Fig. S12A). In addition, BMDCs were pretreated with TLR1/2 inhibitor (C29) and TLR7/9 inhibitor (hydroxychloroquine, HCQ). Neither inhibitor caused an obvious reduction in TNF-α production compared with the F_15_-_D_K_10_ group (Supplementary Fig. S12B), suggesting that TLR1/2- and TLR7/9-mediated signaling are unlikely to be major contributors to F_15_-_D_K_10_-induced BMDC activation. These findings, together with the TAK-242 inhibition results, support the involvement of TLR4-associated signaling in mediating DC activation.

**Fig. 4:**
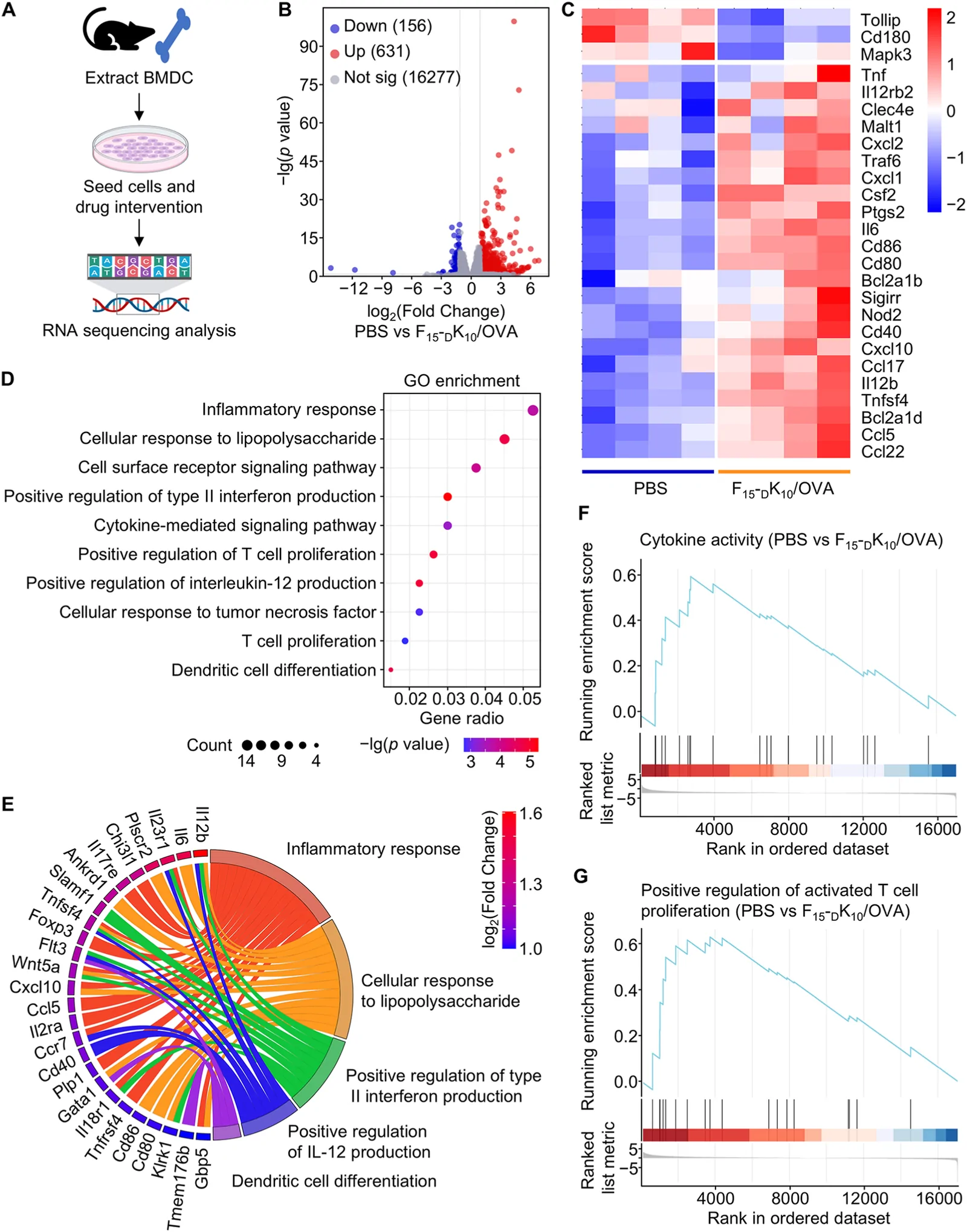
RNA-seq analysis of BMDCs incubated with PBS and F_15_-_D_K_10_/OVA. (A) Experimental strategy for RNA-seq analysis. (B) Volcano plots reveal widespread up-regulation of gene expression. (C) Cluster heatmap showing transcript expression of selected immune-related genes. (D,E) Bubble (D) and chord plots of GO analysis (E) based on differentially expressed genes between PBS and F_15_-_D_K_10_/OVA groups. (F) GSEA of cytokine activity and (G) positive regulation of activated T cell proliferation (*n* = 4 for B−G).

To gain deeper mechanistic insights, RNA sequencing analysis was performed on BMDCs following treatment with PBS or F_15_-_D_K_10_/OVA (Fig. 4A). Compared with the PBS group, F_15_-_D_K_10_/OVA induced marked BMDC activation. Volcano plots analysis demonstrated widespread upregulation of gene expression (Fig. 4B), whereas cluster heatmaps showed pronounced downregulation of *Tollip*, *CD180*, and *Mapk3* (Fig. 4C). These transcriptional changes relieve inhibitory constraints on the TLR4 and nuclear factor-κB (NF-κB) signaling axis, thereby amplifying downstream signaling cascades [41,42]. Reduced expression of *Mapk3* redirects metabolic resources from cellular proliferation toward maturation processes [43]. Concurrently, core pro-inflammatory genes, including *Il12b*, *Il6*, *Tnf*, and *Csf2*, as well as transcripts encoding co-stimulatory molecules *Cd40*, *Cd80*, and *Cd86*, were upregulated, thereby facilitating subsequent T cell activation. Additionally, transcripts associated with chemokine signaling, such as *Ccl5* and *Cxcl10*, and immune-sensing or signal amplification, including *Nod2*, *Traf6*, and *Malt1*, were upregulated [[44], [45], [46], [47]]. Meanwhile, the expression of *SIGIRR* and *Bcl2a1* supported DC survival and homeostasis *via* preventing excessive inflammatory damage [[48], [49], [50]]. Gene Ontology (GO) analysis visualized using bubble and chord plots indicated that F_15_-_D_K_10_/OVA induced a lipopolysaccharide-like stimulus, enhanced IL-12, TNF-α, and IFN-γ secretion, and increased surface receptor expression, collectively promoting proliferation and activation of T cells (Fig. 4D and E). Gene Set Enrichment Analysis (GSEA) further demonstrated marked enrichment of pathways associated with cytokine secretion and T cell proliferation in the F_15_-_D_K_10_/OVA group (Fig. 4F and G). Based on RNA sequencing results, activation of the TLR4−MyD88−NF-κB signaling pathway was further validated by Western blotting containing TLR4, MyD88, NF-κB p65, and NF-κB pp65 proteins (Fig. S13, Supplementary data). While our data suggest that the TLR4−MyD88−NF-κB pathway is the predominant activation pathway for F_15_-_D_K_10_-induced DC maturation, this interpretation does not exclude the potential involvement of additional immune-sensing mechanisms beyond the scope of current investigation.

### 3.4 Biodistribution and immune activation of F-_D_K/OVA in draining lymph nodes

**Fig. 5:**
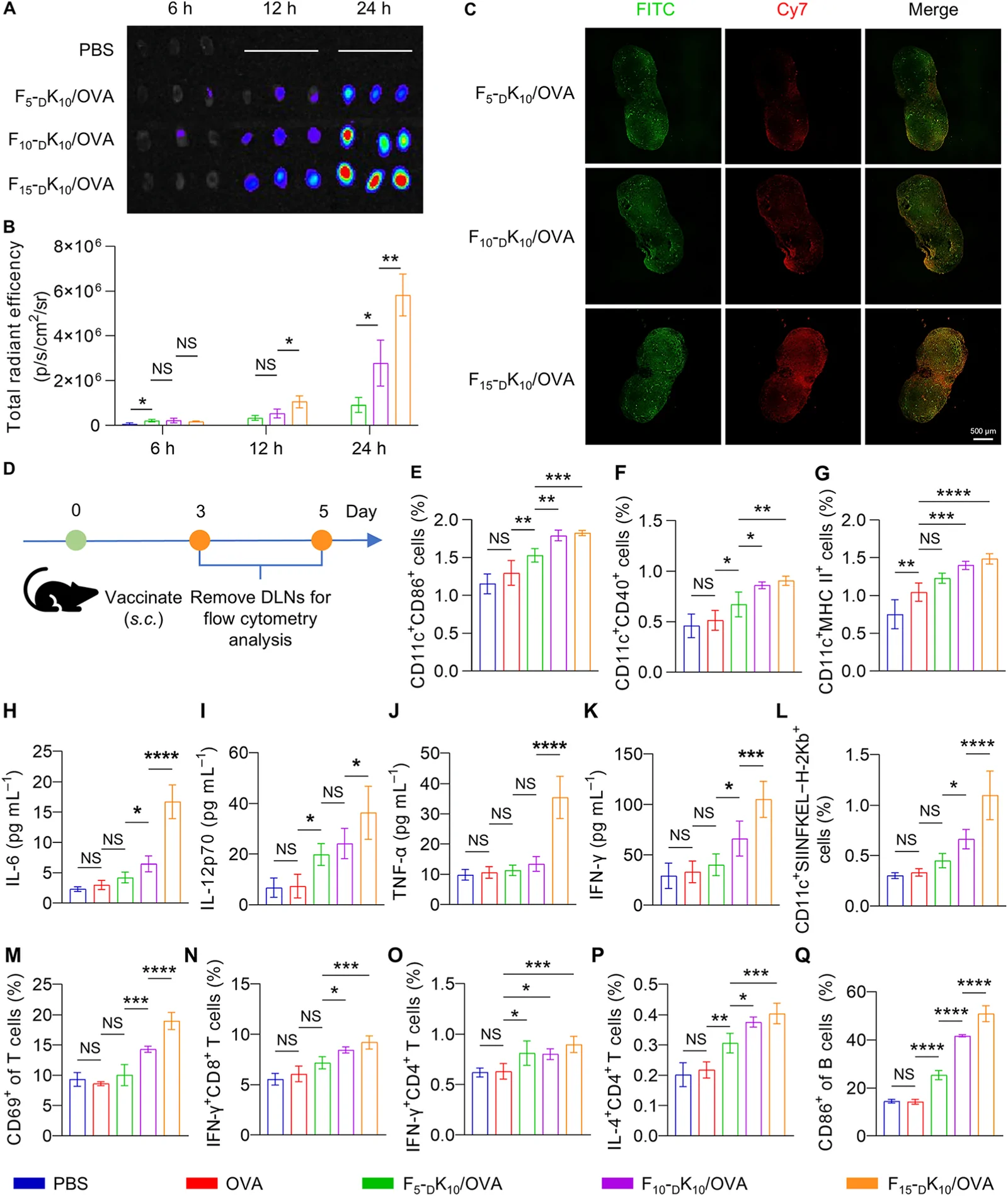
*In vivo* distribution and immunological effects of F-_D_K/OVA. (A,B) Fluorescence imaging (A) and quantitative analysis of inguinal DLNs (B) after *s.c.* injection of Cy7-labeled vaccines into the right footpad at 6, 12, and 24 h. (C) CLSM images showing DC and vaccine biodistribution in DLNs at 24 h after different treatments. CD11c (green) and OVA−Cy7 (red). (D) Experimental strategy of DLN immune response. (E−G) CD11c^+^CD86^+^ (E), CD11c^+^CD40^+^ (F), and CD11c^+^MHC II^+^ cells (G) on day 3 DLNs after different treatments. (H−K) The concentrations of IL-6 (H), IL-12p70 (I), TNF-α (J), and IFN-γ (K) in the DLN homogenate supernatants with different treatments on day 3. (L−Q) CD11c^+^SIINFEKL−H-2Kb^+^ cells (L), CD69^+^ of T cells (M), IFN-γ^+^CD8^+^ T cells (N), Th1 (IFN-γ^+^CD4^+^ T) cells (O), Th2 (IL-4^+^CD4^+^ T) cells (P), and CD86^+^ of B cells (Q) on day 5 DLNs after different treatments. All statistical data are represented as mean ± SD (*n* = 3 for A, *n* = 6 for E−L, *n* = 5 for M−Q; NS, not significant; *P < 0.05, **P < 0.01, ***P < 0.001, ****P < 0.0001).

LNs serve as essential immune sites for initiating adaptive immune responses. Following *in vitro* validation, the ability of F-_D_K/OVA to reach DLNs and activate resident immune cells was evaluated *in vivo*. The *s.c.* administration of F-_D_K/OVA−Cy7 into the footpad resulted in efficient trafficking to LNs, with strong fluorescence retained at 24 h, as observed *via ex vivo* fluorescence imaging. Increased hydrophobicity further enhanced LN accumulation and prolonged retention (Fig. 5A and B). Immunofluorescence staining of DLN sections confirmed clear colocalization of F-_D_K/OVA with CD11c^+^ DCs (Fig. 5C), indicating that the vaccines reached DLNs and were internalized *via* resident DCs. FCM analysis of DLNs further revealed antigen uptake *via* DCs, macrophages, T cells, and B cells (Fig. S14, Supplementary data), suggesting that multiple antigen-presenting cell subsets collectively contribute to antigen presentation and enhance the probability of T cell priming. In addition to evaluating vaccine distribution in DLNs, a systemic biodistribution experiment was conducted. Following *s.c.* injection of F-_D_K/OVA−Cy5 at −12, −24, and −48 h, fluorescence signals in major organs were measured (Fig. S15A and B, Supplementary data). The liver showed the highest signal intensity, followed by the kidney and lung, with a decline observed by 48 h. The liver/DLNs fluorescence intensity ratios at 12 , 24 , and 48 h post *s.c.* injection were 68.19, 92.29, and 101.13, respectively. The pronounced liver-associated signal is nevertheless biologically plausible. Following *s.c.* administration, nano carriers that escape retention in regional LNs enter the systemic circulation through lymphatic drainage and subsequently undergo hepatic clearance [[51], [52], [53]]. Nevertheless, the hepatic accumulation warrants careful safety evaluation. To address this issue, supplementary quantitative tissue biodistribution experiments were performed (Fig. S15C, Supplementary data). The fluorescence-equivalent percent injected dose per gram of liver tissue reached 4.32 ± 0.52%ID/g at 24 h post-administration. Combined with the cytotoxicity data *in vitro*, this level of accumulation falls within a biocompatible range.

Immune activation in DLNs was subsequently assessed. DLNs were excised on days 3 and 5 after *s.c.* injection, and the activation status of DCs, T cells, and B cells was evaluated (Fig. 5D). On day 3, expression of DC maturation markers, including CD86, CD40, and MHC II, increased markedly in a hydrophobicity-dependent manner (Fig. 5E−G). Analysis of cytokines in LN homogenates revealed similar hydrophobicity-dependent increases in IL-6, IL-12p70, TNF-α, and IFN-γ(Fig. 5H−K). On day 5, DC antigen cross-presentation and activation of T cells and B cells were examined. Detailed gating strategies for FCM are provided in Supplementary Figs. S16, S17A, S17B and S18A. The proportion of CD11c^+^SIINFEKL−H-2Kb ^+^ cells in the F_15_-_D_K_10_/OVA group was 1.65-, 2.44-, and 3.28-fold higher than those observed in the F_10_-_D_K_10_/OVA, F_5_-_D_K_10_/OVA, and OVA groups, respectively (Fig. 5L). Early T cell activation, measured *via* CD69 expression, showed a similar hydrophobicity-dependent trend, with proportions in the F_15_-_D_K_10_/OVA group exceeding those in the F_10_-_D_K_10_/OVA, F_5_-_D_K_10_/OVA, and OVA groups by 1.33-, 1.89-, and 2.20-fold, respectively (Fig. 5M). CTLs, defined as IFN-γ^+^CD8^+^ T cells, increased by 1.18-, 1.39-, and 1.51-fold in the F_5_-_D_K_10_/OVA, F_10_-_D_K_10_/OVA, and F_15_-_D_K_10_/OVA groups relative to the OVA group (Fig. 5N and Fig. S17B, Supplementary data). Additionally, expansion of the T helper 1 (Th1) and Th2 subsets was observed (Fig. 5O and P). Notably, activation of B cells also showed hydrophobicity dependence, with CD86^+^ B cells in the F_5_-_D_K_10_/OVA, F_10_-_D_K_10_/OVA, and F_15_-_D_K_10_/OVA groups 1.77-, 2.91-, and 3.55-fold higher than in the OVA group (Fig. 5Q and Fig. S18B, Supplementary data). The concurrent expansion of these subsets reflects a comprehensive immune response that supports tumor clearance through cooperative cellular and humoral mechanisms.

Together, these findings demonstrate delivery to and immune cell engagement within DLNs rather than selective LN targeting. Within the tested formulation series, greater hydrophobic content is associated with higher relative DLN fluorescence and stronger activation of DCs, T cells, and B cells. The coordinated immune responses in DLNs provide a mechanistic basis for the subsequent antigen-specific immune effects.

### 3.5 Efficient tumor therapy with F-_D_K/OVA *in vivo*

**Fig. 6:**
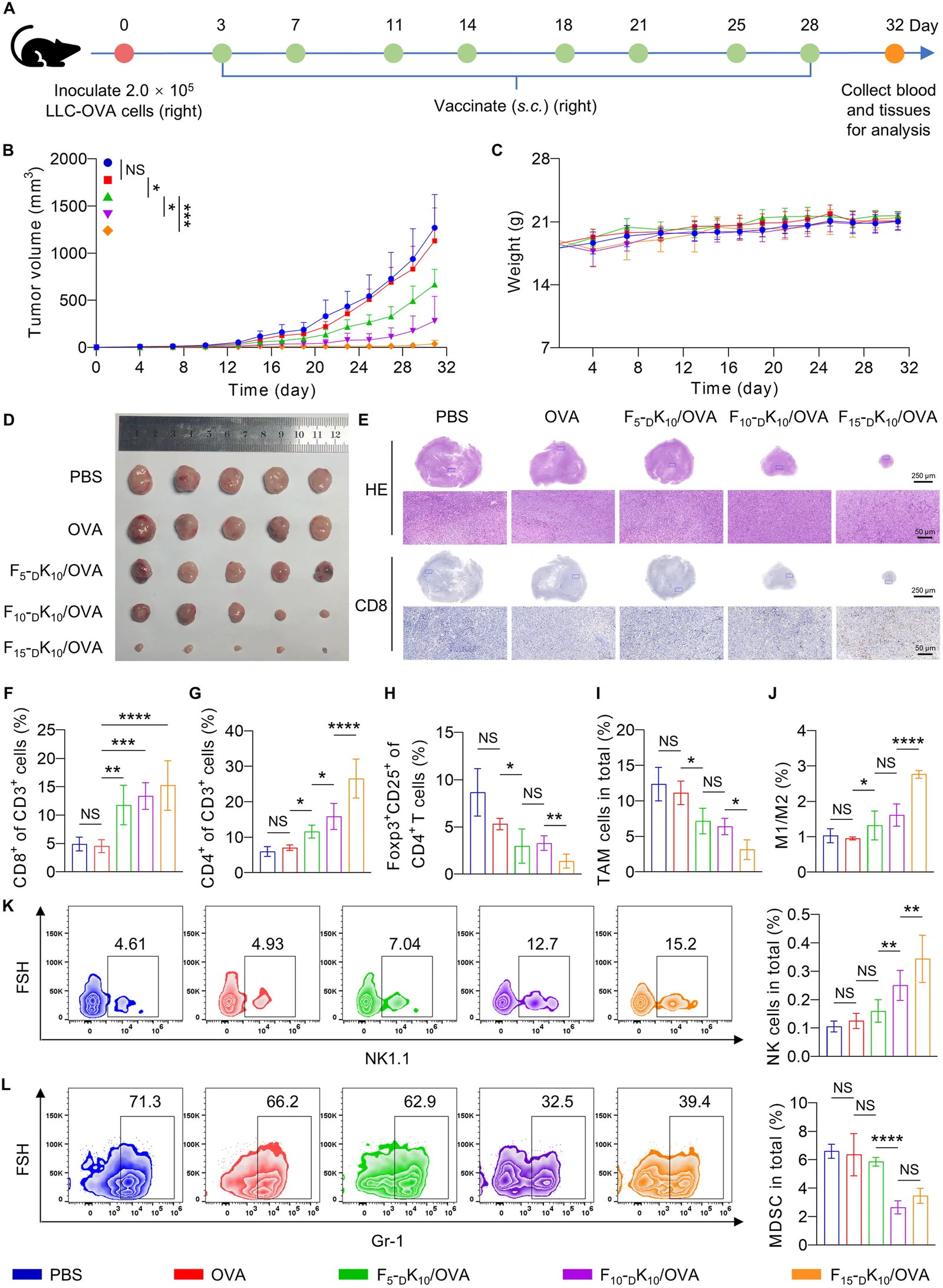
Therapeutic efficacies and immunoregulatory mechanisms of F-_D_K/OVA. (A) Experimental strategy of LLC-OVA treatment study. (B,C) Tumor volumes (B) and body weights of mice (C) receiving different vaccines from day 0−31. (D,E) Representative gross images (D) and H&E and CD8 immunohistochemistry images of tumors (E) after different treatments on day 31. (F−L) CD8^+^ of CD3^+^ cells (F), CD4^+^ of CD3^+^ cells (G), Foxp3^+^CD25^+^ of CD4^+^ cells (H), TAMs (I), M1/M2 (J), NK cells (K), and MDSCs (L) in tumors after different treatments on day 31. All statistical data are represented as mean ± SD (*n* = 7 for A and B, *n* = 5 for D−L; NS, not significant; *P < 0.05, **P < 0.01, ***P < 0.001, ****P < 0.0001).

After confirmation of potent immune activation induced by F-_D_K/OVA *in vitro* and *in vivo*, the therapeutic potential was assessed in tumor-bearing mice. Clinically validated immunotherapies, including programmed cell death protein-1 (PD-1) antibodies, antibodies against cytotoxic T lymphocyte-associated protein 4, and antagonists of lymphocyte activation gene 3, often show limited efficacy in immunologically “cold” tumors characterized by poor T cell infiltration and impaired antigen presentation. To determine whether F-_D_K/OVA could reprogram TIMEs and improve therapeutic response in “cold” tumors, a lung adenocarcinoma model was employed. LLC-OVA was selected as the model system. Mice were subcutaneously injected with 2.0 × 10^5^ tumor cells and randomly assigned to five treatment groups. Once solid tumors became palpable, the indicated treatments were administered (Fig. 6A). Tumor growth was initially slow but accelerated rapidly after day 14. Compared with the PBS group, the F_5_-_D_K_10_/OVA, F_10_-_D_K_10_/OVA, and F_15_-_D_K_10_/OVA groups showed superior anti-tumor activity, with tumor inhibition rates of 31.62%, 65.35%, and 97.81%, respectively (Fig. 6B and Fig. S19, Supplementary data). Body weight remained stable throughout repeated dosing, indicating no overt treatment-associated weight loss of F-_D_K/OVA (Fig. 6C).

**Fig. 7:**
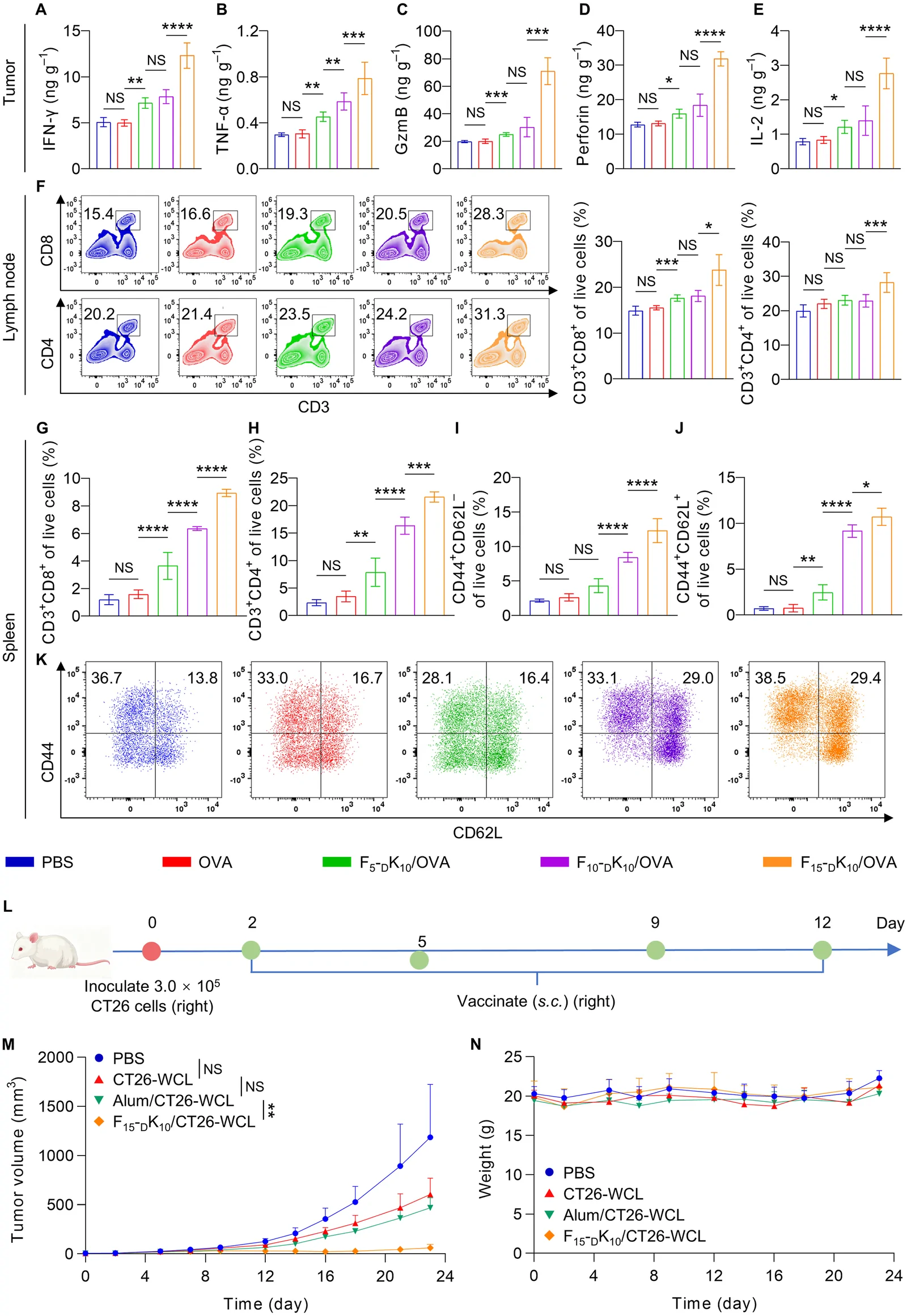
Anti-tumor immunomodulatory mechanisms underlying F-_D_K/OVA. (A−E) Concentrations of IFN-γ (A), TNF-α (B), GzmB (C), perforin (D), and IL-2 (E) in tumor homogenates from different vaccine groups. (F) CD3^+^CD8^+^ and CD3^+^CD4^+^ cells in DLNs after different treatments on day 31. (G−K) CD3^+^CD8^+^ cells (G), (H) CD3^+^CD4^+^ cells (H), CD44^+^CD62L^−^ cells (I, K), and CD44^+^CD62L^+^ (J, K) cells in the spleen after different treatments on day 31. (L) Experimental strategy of the CT-26 treatment study. (M,N) Tumor volumes (M) and body weights of mice (N) receiving different vaccines in the CT26 model from day 0−23. All statistical data are represented as mean ± SD (*n* = 4−5 for A−E, *n* = 5 for F−N; NS, not significant; *P < 0.05, **P < 0.01, ***P < 0.001, ****P < 0.0001).

To elucidate underlying mechanisms, tumors were excised for gross imaging, weight measurement, H&E staining, CD8 immunohistochemistry, and FCM analysis of intratumoral immune subsets. Gross images and tumor weights provided direct visual and quantitative evidence of the superior anti-tumor efficacy of F-_D_K/OVA (Fig. 6D and Fig. S20, Supplementary data). H&E staining of tumors from the PBS group revealed high proliferation, poor differentiation, active mitosis, and pronounced pleomorphism at high magnification (Fig. 6E). In contrast, tumors treated with F-_D_K/OVA, particularly those from the F_15_-_D_K_10_/OVA group, showed markedly reduced mitotic activity and decreased pleomorphism. Immunohistochemical staining for CD8 demonstrated sparse, peripherally localized CD8^+^ T cells in PBS-treated tumors, whereas F-_D_K/OVA induced dense, widespread infiltration of CD8^+^ T cells throughout tumor tissues (Fig. 6E). FCM analysis further confirmed broad immune reprogramming within tumor microenvironments (TMEs). Detailed gating strategies for FCM are provided in Supplementary Figs. S21−S23. Treatment with F-_D_K/OVA increased frequencies of cytotoxic CD8^+^ T cells (4.93%, 4.56%, 11.78%, 13.34%, and 15.24% in the group of PBS, OVA, F_5_-_D_K_10_/OVA, F_10_-_D_K_10_/OVA, and F_15_-_D_K_10_/OVA, respectively), helper CD4^+^ T cells (6.00%, 7.07%, 11.62%, 15.88%, and 26.58%), and NK cells (0.11%, 0.13%, 0.16%, 0.25%, and 0.34%), while reducing immunosuppressive Tregs (8.66%, 5.30%, 2.99%, 3.29%, and 1.39%) and MDSCs (6.62%, 6.39%, 5.89%, 2.66%, and 3.46%). Although overall levels of TAM (12.38%, 11.16%, 7.16%, 6.41%, and 3.15%) declined, without implying a detrimental effect, the ratio of M1 to M2 (1.03, 0.96, 1.32, 1.62, and 2.77%) increased markedly, indicating a favorable anti-tumor immune shift (Fig. 6F−L). Cytokine analysis of tumor homogenates revealed marked increases in IFN-γ, TNF-α, GzmB, perforin, and IL-2 (Fig. 7A−E). In the F_15_-_D_K_10_/OVA group, concentrations of these cytokines increased by 2.46-, 2.58-, 3.54-, 2.43-, and 3.30-fold, respectively, relative to the OVA group.

Analysis of immune parameters in the DLNs and spleens revealed higher proportions of CD4^+^ and CD8^+^ T cells in F-_D_K/OVA groups compared with the OVA group (Fig. 7F−H). This increase is attributed to the vigorous immunostimulatory activity of F-_D_K/OVA and to additional tumor-derived antigens reaching the LNs and spleen *via* lymphatic or blood circulation, thereby promoting secondary expansion of the systemic T cell pools. FCM assays revealed that frequencies of T_EM_ (CD44^+^CD62L^−^) in splenic tissues increased from 2.65% in the OVA group to 4.32%, 8.45%, and 12.30% in the F_5_-_D_K_10_/OVA, F_10_-_D_K_10_/OVA, and F_15_-_D_K_10_/OVA groups, respectively (Fig. 7I). T_CM_ in the spleen increased by 3.26-, 12.07-, and 14.11-fold (Fig. 7J and K). These results indicate that F-_D_K/OVA induces a strong local anti-tumor response and establishes a recirculating, systemic immune memory, thereby providing a cellular basis for the suppression of distant metastasis and recurrence. Serum biochemistry, containing ALT, AST, BUN, and CRE, and serum inflammatory factors remained normal after eight *s.c.* injections (Fig. S24, Supplementary data). Histological analysis of major organs, including the heart, liver, spleen, lung, and kidney, also revealed no pathological lesions (Fig. S25−S29, Supplementary data). Together with the stable body weight, these findings support the absence of overt toxicity at the tested dose and treatment schedule. However, they do not exclude potential subclinical, cell type-specific, cumulative, or delayed effects, particularly those associated with hepatic accumulation. Longer-term and more comprehensive toxicity evaluation will therefore be required to fully assess the safety profile of F-_D_K/OVA for future translational applications.

The LLC-OVA model provides a convenient and well-established platform for evaluating vaccine-induced immune responses. However, OVA is a highly immunogenic exogenous model antigen and therefore cannot fully represent endogenous tumor antigens or neoantigens. To further evaluate the general applicability of F_15_-_D_K_10_ under more physiologically relevant conditions, whole-tumor antigens were prepared from heat-shocked, freeze-thawed CT26 cells using the method reported by Gleisner et al. [54]. The resulting whole cell lysates from CT26 cells (CT26-WCL) were formulated with either F_15_-_D_K_10_ or Alum as the adjuvant and evaluated in a therapeutic CT26 tumor model. The results revealed that the tumor inhibition rate of Alum adjuvant was 60.71%, whereas the F_15_-_D_K_10_-adjuvanted vaccine achieved 95.25%, with no significant change in weight (Fig. 7L–N). These findings indicate that the potent immunostimulatory activity of F_15_-_D_K_10_ extends beyond the OVA model antigen. However, evaluation in a single additional CT26 whole-cell-lysate model is insufficient to establish broad anti-tumor applicability across tumor types, antigen classes, or host settings.

Collectively, the F_15_-_D_K_10_ vaccine remodels TIMEs and converts immunologically “cold” tumors into “hot” tumors in the LLC-OVA model. Despite these encouraging results, the present study remains limited by the use of a relatively small number of preclinical tumor models. Future studies incorporating additional endogenous antigen-based tumor models, spontaneous tumor models, and patient-derived tumor models will be valuable to evaluate further the universality and translational potential of this adjuvant platform.

### 3.6 Immune memory and long-term tumor prevention with F-_D_K/OVA *in vivo*

**Fig. 8:**
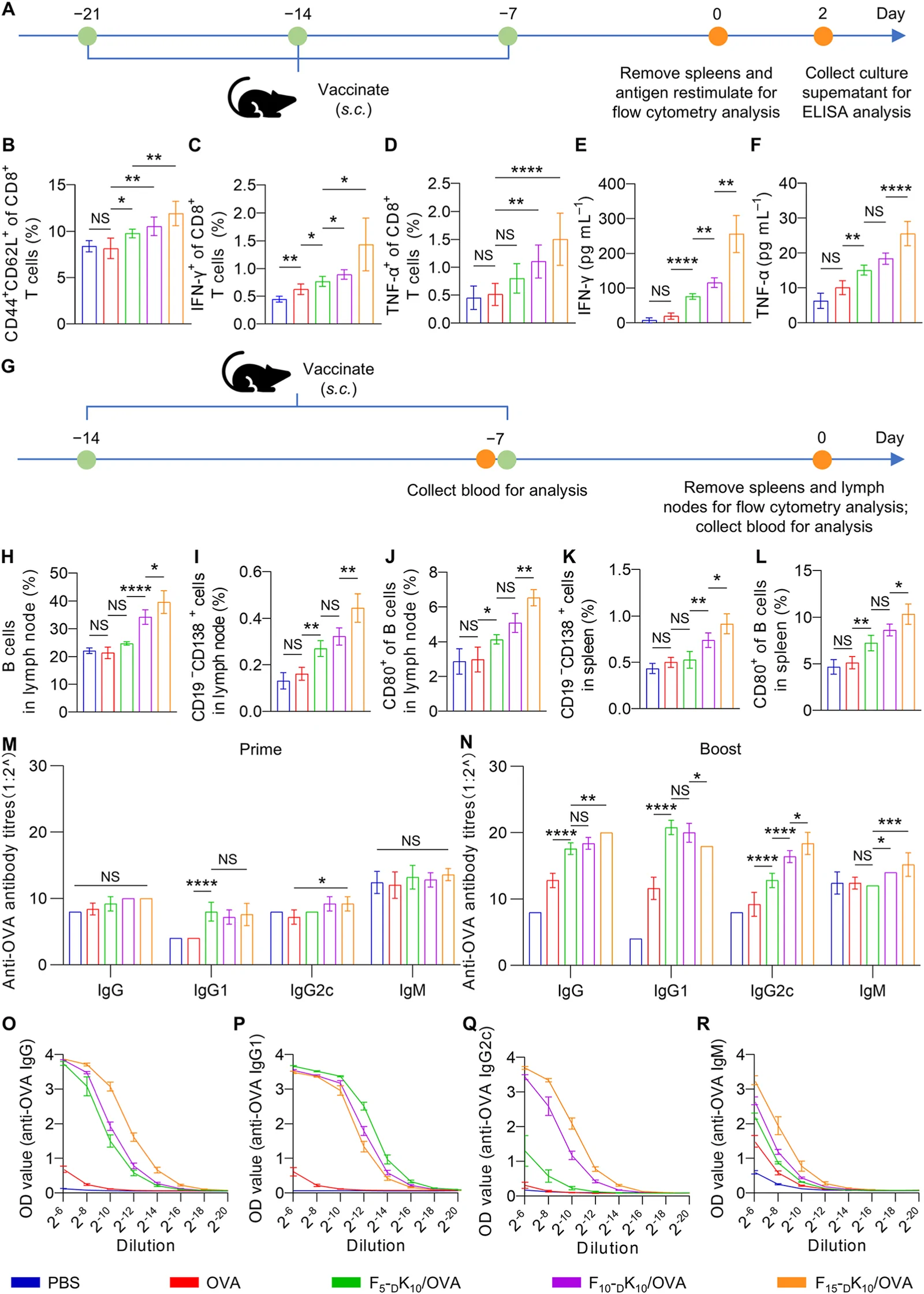
Cellular and humoral immune memory of F-_D_K/OVA *in vivo*. (A) Experimental strategy of cellular immune memory in the spleen. (B) CD44^+^CD62L^+^ of CD8^+^ T cells in the spleen after various treatments. (C,D) IFN-γ^+^ (D) and TNF-α^+^ of CD8^+^ T cells (D) in splenic cells restimulated with OVA_257−264_. (E,F) Concentrations of IFN-γ (E) and TNF-α (F) in the supernatant of restimulated splenic cells. (G) Experimental strategy of humoral immune memory in the DLN, spleen, and blood. (H−J) B cells (H), CD19^−^CD138^+^ cells (I), and CD80^+^ of B cells (J) in the DLN after different treatments on day 0. (K,L) CD19^−^CD138^+^ cells (K) and CD80^+^ of B cells (L) in the spleen after different treatments on day 0. (M) Anti-OVA antibody titers in the serum of mice on day −7 after prime immunization. (N) Anti-OVA antibody titers in the serum of mice on day 0 after boost immunization. (O−R) OD values of anti-OVA IgG (O), anti-OVA IgG1 (P), anti-OVA IgG2c (Q), and anti-OVA IgM (R) in the serum on day 0 after boost immunization. All statistical data are represented as mean ± SD (*n* = 5 for B−F, H−R; NS, not significant; *P < 0.05, **P < 0.01, ***P < 0.001, ****P < 0.0001).

FCM analysis of the therapeutic model above revealed clear potential for durable antigen-specific immune memory, with significant increases in both central and effector memory cells. To further explore the capacity of F-_D_K/OVA to establish immune memory at the cellular and humoral levels, a splenocyte re-stimulation assay was performed to assess cellular immune memory, as well as antibody response and memory B cell analysis for humoral immune memory. After three sequential *s.c.* injections on days −21, −14, and −7, the spleens were harvested on day 0 and analyzed for expression of memory markers CD44 and CD62L *via* FCM. Detailed gating strategies for FCM are provided in Supplementary Fig. S30. Splenocytes were subsequently cultured and restimulated *in vitro* with OVA_257−264_. OVA-specific T cell activation was evaluated by quantifying IFN-γ^+^CD8^+^ and TNF-α^+^CD8^+^ T cells, together with measurement of IFN-γ and TNF-α concentrations in culture supernatants (Fig. 8A). Detailed gating strategies for FCM are provided in Supplementary Fig. S31. Compared with the OVA group, the F_5_-_D_K_10_/OVA, F_10_-_D_K_10_/OVA, and F_15_-_D_K_10_/OVA groups exhibited 1.20-, 1.29-, and 1.46-fold increases in T_CM_ (CD44^+^CD62L^+^) in CD8^+^ T cells, respectively (Fig. 8B). Upon antigen re-stimulation, IFN-γ^+^CD8^+^ T cells increased by 1.22-, 1.43-, and 2.28-fold, whereas IFN-γ concentrations increased by 3.84-, 5.86-, and 12.90-fold in the F_5_-_D_K_10_/OVA, F_10_-_D_K_10_/OVA, and F_15_-_D_K_10_/OVA groups, respectively. TNF-α^+^CD8^+^ T cells and TNF-α concentrations also increased with the optimization of the quantity of hydrophobic moieties (Fig. 8C−F).

To validate humoral immune memory, mice were subcutaneously immunized twice on day −14 (prime) and day −7 (boost). On day 0, cells of the DLNs and spleens were collected to analyze B cell activation, plasma cell formation, and memory B cell frequencies. Serum samples from day −7 (post-prime) and day 0 (post-boost) were harvested to measure anti-OVA antibody responses (Fig. 8G). After two *s.c.* injections, a robust germinal center reaction was observed in LNs, accompanied by increased B cells. The levels of CD19^+^ B cells are 22.10%, 21.38%, 24.78%, 34.26%, and 39.60% in the PBS, OVA, F_5_-_D_K_10_/OVA, F_10_-_D_K_10_/OVA, and F_15_-_D_K_10_/OVA groups, respectively (Fig. 8H). The activation level (CD40^+^) of B cells in DLN increased with hydrophobic moieties of F-_D_K (Fig. S32A, Supplementary data). Plasma cells (CD19^−^CD138^+^), the main source of antibody responses, showed 1.67-fold, 1.99-fold, and 2.74-fold increases in the F_5_-_D_K_10_/OVA, F_10_-_D_K_10_/OVA, and F_15_-_D_K_10_/OVA groups, respectively, compared with the OVA group (Fig. 8I). Similarly, memory B cells (CD80^+^CD19^+^), which are central to long-term humoral immunity, exhibited the similar fold increases (1.39, 1.70, and 2.19, respectively) as hydrophobic moieties of F-_D_K increased (Fig. 8J). In addition to LNs, the spleen is also a key site for humoral immunity. Activated B cells, plasma cells, and memory B cells in the spleen exhibit a consistent trend: Their levels increase as the number of hydrophobic moieties on F-_D_K increases. In the F_15_-_D_K_10_/OVA group, the levels of CD40^+^ on B cells, CD86^+^ on B cells, CD19^−^CD138^+^ cells, and CD80^+^ on B cells are 1.38, 2.38, 1.73, and 1.43 times those in the F_5_-_D_K_10_/OVA group, respectively (Fig. S32B and S32C, Supplementary data) (Fig. 8K and L). On day −7 (post-prime), anti-OVA IgG, IgG1, IgG2c, and IgM titers were uniformly low (Fig. 8M). On day 0 (post-boost), anti-OVA IgG, IgG1, IgG2c, and IgM increased from 10.0 to 20.0, from 7.6 to 18.0, from 9.2 to 18.4, and from 13.6 to 15.2 in the F_15_-_D_K_10_/OVA group (Fig. 8M−R). These results demonstrated that primary immunization (day −14) successfully established immune memory, enabling rapid mobilization of memory B cells and production of higher antibody levels after the second dose. The higher IgG2c levels further indicated that F_15_-_D_K_10_/OVA not only induced humoral immunity but also strongly activated Th1 cellular immunity, a crucial feature of tumor vaccines because Th1 responses facilitate CTL activation and long-term immune surveillance. In addition, although IgM levels also increased, the increases in IgG and its subclasses were substantially greater than those in IgM, indicating that B cells underwent effective affinity maturation and class switching in germinal centers, producing high-affinity mature antibodies.

**Fig. 9:**
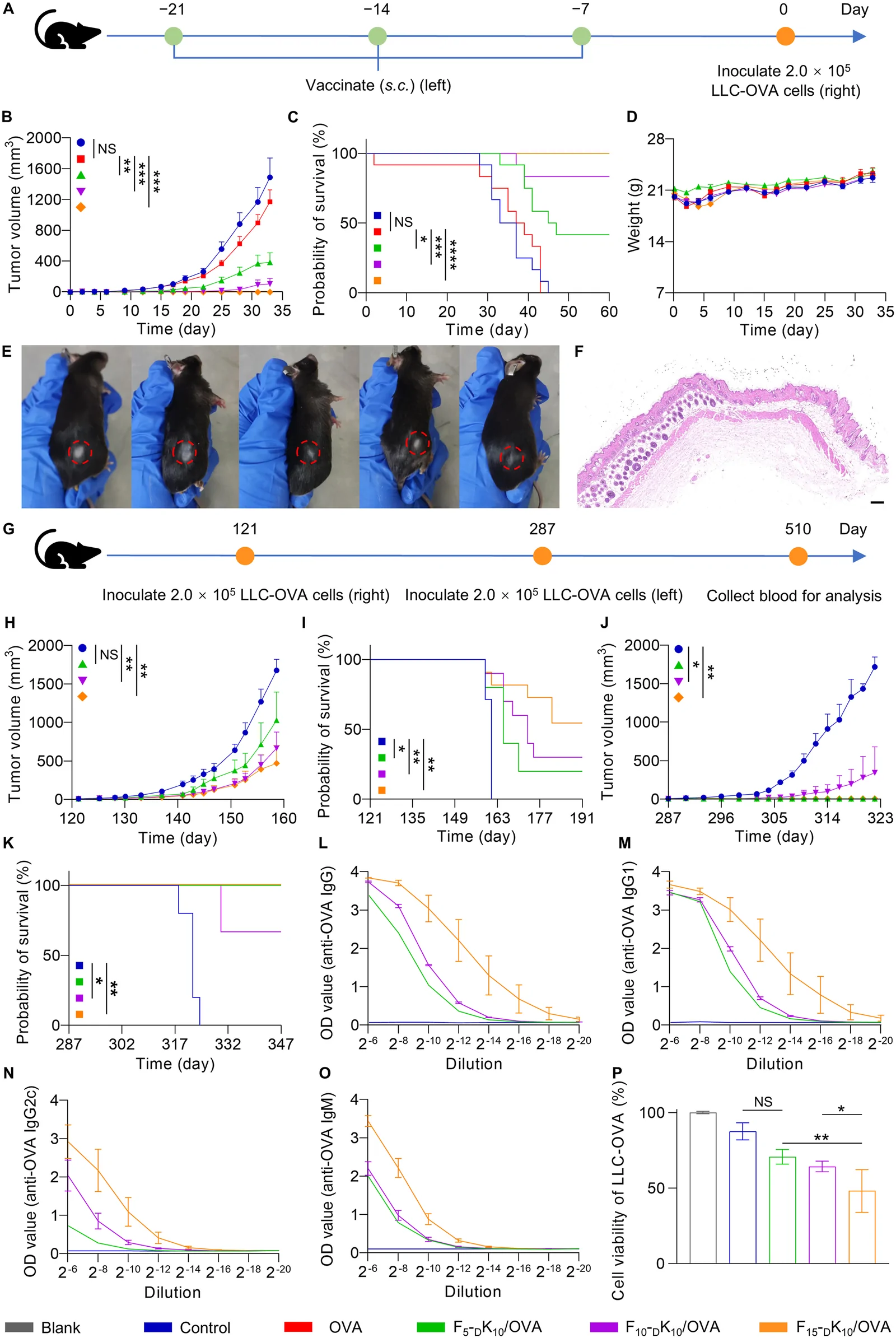
Elevating prevention efficiency of F-_D_K/OVA. (A) Experimental strategy of the first LLC-OVA prevention model. (B−D) Tumor volumes (B), probabilities of survival (C), and weights of mice (D) receiving various vaccines from day 0−60. (E) Faint scars at the tumor site in tumor-free mice receiving F-_D_K/OVA after the first tumor challenge. (F) H&E images of skin from a tumor-free mouse after the first tumor challenge. Scale bar: 200.0 μm. (G) Experimental strategy of the second and third LLC-OVA prevention model. (H,I) Tumor volumes (H) and probabilities of survival of mice (I) receiving various vaccines from day 121−191. (J,K) Tumor volumes (J) and probabilities of survival of mice (K) receiving various vaccines from day 287−347. (L−O) OD values of anti-OVA IgG (L), anti-OVA IgG1 (M), anti-OVA IgG2c (N), and anti-OVA IgM in the serum of surviving mice (O) on day 510. (P) The cell viability of LLC-OVA cells after incubating with the serum diluted 64-fold and rabbit complement. All statistical data are represented as the mean ± standard error of the mean (SEM; *n* = 12 for B−D, *n* = 7 in Control group from H and I, *n* = 5 in Control group from J−P; NS, not significant; *P < 0.05, **P < 0.01, ***P < 0.001, ****P < 0.0001).

Based on the cellular and humoral immune memory induced by F-_D_K/OVA, a prevention model was established to assess its protective efficacy. Mice were divided into five groups and received three *s.c.* immunizations (Fig. 9A). Tumor challenge was subsequently performed on the contralateral flank to mimic the random tumor initiation *in vivo*. Tumors in the Control and free OVA groups exhibited rapid growth beginning around day 14 (Fig. 9B), reaching 100% incidence at the endpoint. In contrast, a substantial proportion of mice treated with F-_D_K/OVA remained tumor-free (Fig. 9C). Initial protective efficacies reached 41.67%, 83.33%, and 100.00% in the F_5_-_D_K_10_/OVA, F_10_-_D_K_10_/OVA, and F_15_-_D_K_10_/OVA groups, respectively. Body weight remained stable throughout the observation period (Fig. 9D). Scar-like marks were observed at challenge sites in tumor-free mice, but histological analysis showed no residual tumor cells, indicating complete tumor clearance (Fig. 9E and F).

To evaluate the durability of immune memory in the LLC-OVA model, tumor-free mice were subjected to two additional tumor re-challenges (Fig. 9G). Two response patterns were observed: Some mice lost immunity protection and developed tumors comparable to controls, whereas others showed markedly delayed growth or complete resistance, resulting in long-term tumor-free survival (Fig. S33 and S34, Supplementary data). Cumulative protection rates after the second and third tumor challenges reached 0.08%, 16.67%, and 54.55% in the F_5_-_D_K_10_/OVA, F_10_-_D_K_10_/OVA, and F_15_-_D_K_10_/OVA groups, respectively (Fig. 9H−K). Tumor-free mice survived for 644 days, demonstrating sustained long-term anti-tumor efficacy of F-_D_K/OVA in this repeated LLC-OVA challenge model. No obvious body weight loss was observed during the extended study (Fig. S35A and S35B, Supplementary data). Furthermore, H&E staining of major organs, including the heart, liver, spleen, lung, and kidney, from the long-term surviving F_15_-_D_K_10_/OVA-treated mouse on day 644 revealed no obvious organ damage or pathological lesions (Fig. S36, Supplementary data). On day 510, blood from tumor-free mice was collected to assess long-term antibody titers. The results showed that these mice still had high concentrations of anti-OVA IgG, IgG1, IgG2c, and IgM, indicating that our vaccine established and sustainably maintained humoral immune memory for an extended period in the LLC-OVA model (Fig. 9L−O). However, the results also showed that IgG1 titers exceeded IgG2c titers, indicating attenuation of the immune response over time. Subsequently, serum samples were diluted to 1:24 and used in a complement-dependent cytotoxicity assay. The F_5_-_D_K_10_/OVA, F_10_-_D_K_10_/OVA, and F_15_-_D_K_10_/OVA groups were found to kill 29.24%, 35.73%, and 51.87% of LLC-OVA cells, respectively (Fig. 9P). Experiments demonstrated that even 510 days after immunization, antibodies in the serum still effectively recognized the OVA antigen on the surface of tumor cells, successfully activated the complement pathway, and ultimately led to target cell lysis in the LLC-OVA model.

Overall, F-_D_K/OVA induces robust antigen-specific cellular and humoral immune memory. Increasing the hydrophobic content further amplifies secondary responses of memory T cells and B cells within this experimental system, providing a tunable strategy for designing durable and adaptable tumor vaccine adjuvants.

## 4 Conclusion

In this study, amphiphilic cationic block poly(amino acid) adjuvants with varying hydrophobic contents were synthesized and loaded with tumor antigens to achieve potent therapeutic efficacies and durable anti-tumor immunity *via* cellular and humoral immune responses. The therapeutic efficacies demonstrated in the LLC-OVA and CT26 models and the durable immune protection observed in the LLC-OVA repeated-challenge model are confined to the experimental systems investigated here. Increasing the hydrophobic component altered the nanovaccine assembly and regulated multiple physicochemical characteristics, including particle size, zeta potential, and particle rigidity. These physicochemical changes collectively enhanced cellular uptake, DC activation *via* the TLR4-MyD88-NF-κB pathway, and antigen cross-presentation, while prolonging adjuvant retention and promoting uptake by macrophages, B cells, and T cells in LNs. The sequential amplification of these biological processes ultimately elicited potent systemic cellular and humoral immunity in the F_15_-_D_K_10_/OVA group. In the LLC-OVA model, the optimized adjuvant reprogrammed TIMEs, from “cold” to “hot”, by increasing infiltration of tumor-killing cells, including CD8^+^ T and NK cells, while reducing populations of immunosuppressive cells, including Tregs and MDSCs. Notably, F_15_-_D_K_10_/OVA increased both the proportion and persistence of cellular and humoral immune memory, which rapidly generated specific effector lymphocytes and antibody upon tumor challenge and trafficked to the tumor site to reinforce anti-tumor activity. Collectively, these findings highlight the potential of tunable amphiphilic cationic poly(amino acid) adjuvants for effective tumor immunotherapy and durable protection. Increasing the hydrophobicity of the amphoteric cationic polymerization platform enhanced effector function and memory formation in cellular and humoral immunity, providing a robust framework for future vaccine design. Meanwhile, the current study is limited by the absence of lymph-node-targeting capability and by evaluation in a limited number of tumor and antigen models. Future studies involving additional tumor types, antigen systems, and clinically relevant models will be required to determine the broader applicability and translational potential of this adjuvant platform. Further optimization of lymph-node delivery may also improve immune-organ accumulation, reduce nonspecific tissue exposure, and enhance vaccine utilization.

## Ethics approval and consent to participate

All animal studies were performed following relevant ethical regulations, and the procedures were approved by the Institutional Animal Care and Use Committee (IACUC) of the Changchun Institute of Applied Chemistry, Chinese Academy of Sciences (IACUC issue NO. CIAC 2022 [0094]).

## CRediT authorship contribution statement

**Changjuan Qin:** Conceptualization, Formal analysis, Investigation, Methodology, Resources, Validation, Writing – original draft, Writing – review & editing. **Hua Xin:** Conceptualization, Funding acquisition, Supervision. **Qi Wei:** Writing – review & editing. **Qingyang Xiao:** Writing – review & editing. **Guanqing Yang:** Funding acquisition, Investigation, Supervision, Writing – review & editing. **Jianxun Ding:** Conceptualization, Funding acquisition, Project administration, Supervision, Writing – review & editing.

## Declaration of competing interest

The authors declare that they have no known competing financial interests or personal relationships that could have appeared to influence the work reported in this paper.

## References

[1] Li Z., Zhang H., Gong Q., Luo K. (2025). Biomaterials nanoplatform-based tumor vaccines for immunotherapy. Bioact. Mater..

[2] Saxena M., van der Burg S.H., Melief C.J.M., Bhardwaj N. (2021). Therapeutic cancer vaccines. Nat. Rev. Cancer.

[3] Wu J., Liang J., Li S., Lu J., Li Y., Zhang B., Gao M., Zhou J., Zhang Y., Chen J. (2025). Cancer vaccine designed from homologous ferritin-based fusion protein with enhanced DC-T cell crosstalk for durable ddaptive immunity against tumors. Bioact. Mater..

[4] Cheng F., Wang B., Zhu X., Wang Z., Wang H., Li R., Xing C., Ju Y., Wei R., Li X., Meng S. (2026). Alternative antigen retention by a GP96-fusion approach induces long-lasting and broad immunity in mice. Nat. Commun..

[5] Cheng X., Cai H., Li X., Zhang Y., Li S., Li Y., Hu N., Lui S., Jing J., Gong Q., Luo K. (2026). Reversing adenosine-mediated immunosuppression in triple-negative breast cancer by synergistic chemo-immunotherapy *via* stimuli-responsive nanomedicines. EBioMedicine.

[6] Yan W., Cao Y., Xu S., Li Y., Wu T., Yuan W., Yin Q., Li Y. (2024). Personalized multi-epitope nanovaccine unlocks B cell-mediated multiple pathways of antitumor immunity. Adv. Mater..

[7] Ben-Akiva E., Chapman A., Mao T., Irvine D.J. (2025). Linking vaccine adjuvant mechanisms of actionto function. Sci. Immunol..

[8] Xu S., Sun C., Qian T., Chen Y., Dong X., Wang A., Zhang Q., Ji Y., Jin Z., Liu C., Zhao K. (2025). Animal vaccine revolution: Nanoparticle adjuvants open the future of vaccinology. J. Control. Release.

[9] Dai L., Yang L., Nedosekov V., Ma J., Fang W., Feng H., Shu J., He Y. (2026). Advancements in nanomaterial-based adjuvants for animal vaccines. Mater. Today Bio.

[10] Lee S., Kim J., Kim B., Kim B.-s., Shin J., Choi J., Kim M., Seo Y.H., Kim B.H., Lim K.S., Ha S.-J., Choi S.E., Kim H.-O. (2026). Engineering cationic nanovaccines to enable precision immunotherapy in infectious diseases and cancer. Discov. Appl. Sci..

[11] Farooq M.A., Johnston A.P.R., Trevaskis N.L. (2025). Impact of nanoparticle properties on immune cell interactions in the lymph node. Acta Biomater..

[12] Liu X., Min Q., Song H., Yue A., Li Q., Zhou Q., Han W. (2023). Potentiating humoral and cellular immunity using a novel hybrid polymer-lipid nanoparticle adjuvant for HBsAg-VLP vaccine. J. Nanobiotechnol..

[13] Lei H., Alu A., Yang J., He X., He C., Ren W., Chen Z., Hong W., Chen L., He X., Yang L., Li J., Wang Z., Wang W., Wei Y., Lu S., Lu G., Song X., Wei X. (2023). Cationic crosslinked carbon dots-adjuvanted intranasal vaccine induces protective immunity against Omicron-included SARS-CoV-2 variants. Nat. Commun..

[14] Wu J., Ni M., Xing L., Wang Y., Liu X., Zheng Y., Li L., Huang Y. (2026). Surface hydrophobicity and rigidity determines protein Corona on orally delivered nanoparticles treating colitis. Nat. Commun..

[15] Moyano D.F., Goldsmith M., Solfiell D.J., Landesman-Milo D., Miranda O.R., Peer D., Rotello V.M. (2012). Nanoparticle hydrophobicity dictates immune response. J. Am. Chem. Soc..

[16] Malhotra M., Chotaliya D., Debnath M., Patel R., Kulkarni A. (2024). Varying the hydrophobic core composition of polymeric nanoparticles affects NLRP3 inflammasome activation. Biomater. Sci..

[17] Zhang Y., Zhang M., Zheng J., Li Z., Guo Q., Chen Y., Chen Y., Jiang X., Tang J. (2025). Nanocarrier nanomechanics: a key to unlocking endocytosis in cancer cells navigating epithelial–mesenchymal transition dynamics. ACS Nano.

[18] Qin C., Yang G., Wei Q., Xin H., Ding J., Chen X. (2024). Multidimensional role of amino acid metabolism in immune regulation: From molecular mechanisms to therapeutic strategies. Chem. Res. Chin. Univ..

[19] Wei Q., Xin H., Wang X., Qin C., Su Y., Li D., Ding J. (2025). Unimolecular chiral poly(amino acid)s as adjuvants of nanovaccines for augmented cancer immunotherapy. Chin. Chem. Lett..

[20] Wang H., Li H., Wang X., Ding J. (2025). Immunologically effective chiral polymers to potentiate anti-cancer immune responses. Polym. Sci. Technol..

[21] Su Y., Xu W., Wei Q., Ma Y., Ding J., Chen X. (2023). Chiral polypeptide nanoparticles as nanoadjuvants of nanovaccines for efficient cancer prevention and therapy. Sci. Bull..

[22] Lee H. (2024). Recent advances in simulation studies on the protein Corona. Pharmaceutics.

[23] Guo F., Lv Q., Zhao J., Wang J., Chen J., Yu N., Hong W., Lu Y., Yang G. (2025). Charge-driven Corona formation and nanoparticle cellular uptake: A microfluidic study on nanoparticle-protein interactions. Colloids Surf. B Biointerfaces.

[24] Mahmoudi M., Landry M.P., Moore A., Coreas R. (2023). The protein Corona from nanomedicine to environmental science. Nat. Rev. Mater..

[25] Olszewski W.L., Lee B.-B., Bergan J., Rockson S.G. (2011). Lymphedema: A Concise Compendium of Theory and Practice.

[26] Guo W., Zhang X., Wan L., Wang Z., Han M., Yan Z., Li J., Deng R., Li S., Mao Y., Wang S. (2024). β-glucan-modified nanoparticles with different particle sizes exhibit different lymphatic targeting efficiencies and adjuvant effects. J. Pharm. Anal..

[27] Qiao N., Hori M., Naito M., Chang H., Ogura S., Suzuki M., Kimura T., Fukushima S., Kim H.J., Uchida S., Miyata K. (2026). Increasing polycation hydrophobicity in mRNA polyplex vaccines enhances the efficacy of humoral and cellular immunity induction. Biomaterials.

[28] Nakamura T., Kawai M., Sato Y., Maeki M., Tokeshi M., Harashima H. (2020). The effect of size and charge of lipid nanoparticles prepared by microfluidic mixing on their lymph node transitivity and distribution. Mol. Pharm..

[29] Mott L., Akers C., Pack D.W. (2023). Effect of polyplex surface charge on cellular internalization and intracellular trafficking. J. Drug Deliv. Sci. Technol..

[30] Chen J., Fang H., Hu Y., Wu J., Zhang S., Feng Y., Lin L., Tian H., Chen X. (2022). Combining mannose receptor mediated nanovaccines and gene regulated PD-L1 blockade for boosting cancer immunotherapy. Bioact. Mater..

[31] Wei X., Shao B., He Z., Ye T., Luo M., Sang Y., Liang X., Wang W., Luo S., Yang S., Zhang S., Gong C., Gou M., Deng H., Zhao Y., Yang H., Deng S., Zhao C., Yang L., Qian Z., Li J., Sun X., Han J., Jiang C., Wu M., Zhang Z. (2015). Cationic nanocarriers induce cell necrosis through impairment of Na^+^/K^+^-ATPase and cause subsequent inflammatory response. Cell Res..

[32] Lin R., Zhao T., Chen L., Liu M., Yu H., Wang R., Yuan M., Li X., Zhao D. (2024). Amphipathicity mediated endocytosis of mesoporous silica nanoparticles with tunable frameworks. Nano Res..

[33] Li Y., Chen X., Gu N. (2008). Computational investigation of interaction between nanoparticles and membranes: Hydrophobic/hydrophilic effect. J. Phys. Chem. B.

[34] de Araujo A.D., Hoang H.N., Lim J., Mak J.Y.W., Fairlie D.P. (2022). Tuning electrostatic and hydrophobic surfaces of aromatic rings to enhance membrane association and cell uptake of peptides. Angew. Chem. Int. Ed..

[35] Pašalić L., Jakas A., Pem B., Bakarić D. (2023). Adsorption/Desorption of cationic-hydrophobic peptides on zwitterionic lipid bilayer is associated with the possibility of proton transfer. Antibiotics.

[36] Shiba H., Nishio M., Sawada M., Tamaki M., Michigami M., Nakai S., Nakase I., Fujii I., Matsumoto A., Kojima C. (2022). Carboxy-terminal dendrimers with phenylalanine for a pH-sensitive delivery system into immune cells including T cells. J. Mater. Chem. B.

[37] Zhao Y., Song D., Wang Z., Huang Q., Huang F., Ye Z., Wich D., Chen M., Khirallah J., Gao S., Liu Y., Xu Q. (2024). Antitumour vaccination *via* the targeted proteolysis of antigens isolated from tumour lysates. Nat. Biomed. Eng..

[38] Ma J., Wei K., Zhang H., Tang K., Li F., Zhang T., Liu J., Xu P., Yu Y., Sun W., Zhu L., Chen J., Zhou L., Liang X., Lv J., Fiskesund R., Liu Y., Huang B. (2018). Mechanisms by which dendritic cells present tumor microparticle antigens to CD8^+^ T cells. Cancer Immunol. Res..

[39] Wang R., Yin C., Liu C., Sun Y., Xiao P., Li J., Yang S., Wu W., Jiang X. (2021). Phenylboronic acid modification augments the lysosome escape and antitumor efficacy of a cylindrical polymer brush-based prodrug. J. Am. Chem. Soc..

[40] Qiu C., Xia F., Zhang J., Shi Q., Meng Y., Wang C., Pang H., Gu L., Xu C., Guo Q., Wang J. (2023). Advanced strategies for overcoming endosomal/lysosomal barrier in nanodrug delivery. Research.

[41] Edwards K., Lydyard P.M., Kulikova N., Tsertsvadze T., Volpi E.V., Chiorazzi N., Porakishvili N. (2023). The role of CD180 in hematological malignancies and inflammatory disorders. Mol. Med..

[42] Liew F.Y., Xu D., Brint E.K., O'Neill L.A.J. (2005). Negative regulation of toll-like receptor-mediated immune responses. Nat. Rev. Immunol..

[43] Bendix I., Pfueller C.F., Leuenberger T., Glezeva N., Siffrin V., Müller Y., Prozorovski T., Hansen W., Topphoff U.S., Loddenkemper C., Zipp F., Waiczies S. (2010). MAPK3 deficiency drives autoimmunity *via* DC arming. Eur. J. Immunol..

[44] Ma H., Fang W., Li Q., Wang Y., Hou S.X. (2023). Arf1 ablation in colorectal cancer cells activates a super signal complex in DC to enhance anti-tumor immunity. Adv. Sci..

[45] Isay S.E., Vornholz L., Schnalzger T., Groll T., Magg T., Loll P., Weirich G., Steiger K., Hauck F., Ruland J. (2024). Enforced CARD11/MALT1 signaling in dendritic cells triggers hemophagocytic lymphohistiocytosis. Proc. Natl. Acad. Sci. U. S. A..

[46] Bo R., Liu Z., Zhang J., Gu P., Ou N., Sun Y., Hu Y., Liu J., Wang D. (2019). Mechanism of Lycium barbarum polysaccharides liposomes on activating murine dendritic cells. Carbohydr. Polym..

[47] Kobayashi T., Walsh P.T., Walsh M.C., Speirs K.M., Chiffoleau E., King C.G., Hancock W.W., Caamano J.H., Hunter C.A., Scott P., Turka L.A., Choi Y. (2003). TRAF6 is a critical factor for dendritic cell maturation and development. Immunity.

[48] Vogler M. (2011). BCL2A1: The underdog in the BCL2 family. Cell Death Differ..

[49] Cheng S., Cui L., Chen J., Xiu Q., Si R., Yang X., Shi Y. (2025). SIGIRR: An orphan receptor mediating anti-inflammatory actions. Expert Rev. Mol. Med..

[50] Li L., Wei J., Li S., Jacko A.M., Weathington N.M., Mallampalli R.K., Zhao J., Zhao Y. (2019). The deubiquitinase USP13 stabilizes the anti-inflammatory receptor IL-1R8/Sigirr to suppress lung inflammation. EBioMedicine.

[51] Fernandes S., Xu R., De Rose R., Gu Y., Dominicis A., Bhangu S.K., Mahmoudinoodezh H., Zhu H., Forte G., Hagemeyer C.E., Caruso F., Cavalieri F. (2025). Trafficking glycogen nanoparticles through lymph node tissues for the delivery of small and large bioactive molecules. ACS Nano.

[52] Zeng Y.C., Young O.J., Wintersinger C.M., Anastassacos F.M., MacDonald J.I., Isinelli G., Dellacherie M.O., Sobral M., Bai H., Graveline A.R., Vernet A., Sanchez M., Mulligan K., Choi Y., Ferrante T.C., Keskin D.B., Fell G.G., Neuberg D., Wu C.J., Mooney D.J., Kwon I.C., Ryu J.H., Shih W.M. (2024). Fine tuning of CpG spatial distribution with DNA origami for improved cancer vaccination. Nat. Nanotechnol..

[53] Li N., Qin H., Zhu F., Ding H., Chen Y., Lin Y., Deng R., Ma T., Lv Y., Xiong C., Li R., Wei Y., Shi J., Chen H., Zhao Y., Zhou G., Guo H., Lv M., Lin Y., Han B., Nie G., Zhao R. (2024). Potent prophylactic cancer vaccines harnessing surface antigens shared by tumour cells and induced pluripotent stem cells. Nat. Biomed. Eng..

[54] Gleisner M.A., Pereda C., Tittarelli A., Navarrete M., Fuentes C., Ávalos I., Tempio F., Araya J.P., Becker M.I., González F.E., López M.N., Salazar-Onfray F. (2020). A heat-shocked melanoma cell lysate vaccine enhances tumor infiltration by prototypic effector T cells inhibiting tumor growth. J. Immunother. Cancer.

