# Supplementary material for: Increasing hydrophobicity in poly(amino acid) adjuvants elicits potent and durable anti-tumor immunity via coordinated cellular and humoral responses
Source: Bioact Mater. 2026 Aug 19;67:541–59. doi: 10.1016/j.bioactmat.2026.08.009 (PMC13520902; doi:10.1016/j.bioactmat.2026.08.009)
Supplement: Supplementary file 1 [file mmc1.docx]

**Supplementary data**

**Increasing hydrophobicity in poly(amino acid) adjuvants elicits potent and** **durable anti-tumor immunity *via*** **coordinated cellular and humoral responses**

Changjuan Qin ^a,b^, Hua Xin ^a,^*, Qi Wei ^a,b^, Qingyang Xiao ^b,c^, Guanqing Yang ^b,^*, Jianxun Ding ^b,c,^*

^a^ *Department of Thoracic Surgery, China-Japan Union Hospital of Jilin University, 126 Xiantai Street, Changchun 130033, P. R. China*

^b^ *State Key Laboratory of Polymer Science and Technology, Changchun Institute of Applied Chemistry, Chinese Academy of Science, 5625 Renmin Street, Changchun 130022, P. R. China*

^c^ *School of Applied Chemistry and Engineering, University of Science and Technology of China, 96 Jinzhai Road, Hefei 230026, P. R. China*

* Corresponding authors.

*E-mail addresses:* xhua@jlu.edu.cn (H. Xin), gqyang71@ciac.ac.cn (G. Yang), jxding@ciac.ac.cn (J. Ding).

Materials and methods

*1. Materials*

*N*(ε)-benzyloxycarbonyl-D-lysine (Cbz-D-Lys), L-phenylalanine (L-Phe), tetrahydrofuran, triphosgene, *N*,*N*-dimethylformamide (DMF), hydrogen bromide (33 wt% solution in acetic acid; HBr/HAc), methanol, and fluorescein isothiocyanate (FITC) were purchased from Shanghai Aladdin Biochemical Technology Co., Ltd. (Shanghai, P. R. China). Tribromoethanol and puromycin were purchased from Shanghai Adamas Reagent Co., Ltd. (Shanghai, P. R. China). *n*-Hexylamine (*n*-HA) and ovalbumin (OVA) were purchased from Shanghai Sigma-Aldrich Trading Co., Ltd. (Shanghai, P. R. China). Trifluoroacetic acid (TFA) was purchased from Shanghai Meryer Chemical Technology Co., Ltd. (Shanghai, P. R. China). Deuterated chloroform (CDCl_3_) and deuterated trifluoroacetic acid (TFA-*d*) were purchased from Shanghai Energy Chemical Technology Co., Ltd. (Shanghai, P. R. China).

Roswell Park Memorial Institute 1640 medium (RPMI 1640), Dulbecco's modified Eagle's medium (DMEM), 100 × penicillin/streptomycin (P/S) solution, 1× phosphate-buffered saline (PBS), 4.0% (*W*/*V*) paraformaldehyde (PFA), phenylmethanesulfonyl fluoride (PMSF), phosphatase inhibitor (PI), 50 × cocktail, RIPA lysis buffer, 5 × sodium dodecyl sulfate−polyacrylamide gel electrophoresis (SDS−PAGE) sample loading buffer, 0.45 μm polyvinylidene fluoride (PVDF) membranes, protein-free rapid blocking buffer, recombinant rabbit anti-β-actin antibody, horseradish peroxidase-conjugated goat anti-rabbit immunoglobulin G (IgG; H+L), and enhanced chemiluminescence (ECL) detection kits were purchased from Wuhan Servicebio Technology Co., Ltd. (Hubei, P. R. China). Fetal bovine serum (FBS) was purchased from Zhejiang TianHang Biotechnology Co., Ltd. (Zhejiang, P. R. China). Bicinchoninic acid (BCA) protein assay kits, plasma membrane staining kits with 3,3'-dioctadecyloxacarbocyanine perchlorate (DiO), Hoechst 33342 staining solution, LysoTracker Green, TRIzol reagent, primary antibody dilution buffer, and secondary antibody dilution buffer were purchased from Shanghai Beyotime Biotechnology Co., Ltd. (Shanghai, P. R. China). Cell Counting Kit-8 (CCK-8) was purchased from Dalian Meilun Biotechnology Co., Ltd. (Liaoning, P. R. China). Cyanine 5-*N*-hydroxysuccinimide ester (Cy5-NHS) and Cy7-NHS ester were purchased from Beijing Fluorescence Biotechnology Co., Ltd. (Beijing, P. R. China). SDS−PAGE gel quick preparation kits and 3-color prestained protein markers were purchased from Shanghai Epizyme Biomedical Technology Co., Ltd. (Shanghai, P. R. China).

Rabbit polyclonal antibodies against Toll-like receptor 4 (TLR4), myeloid differentiation primary response protein 88 (MyD88), and nuclear factor-κB (NF-κB) p65 were purchased from Affinity Biosciences Group Ltd. (Jiangsu, P. R. China). Mouse granulocyte-macrophage colony-stimulating factor (GM-CSF) recombinant protein, mouse interleukin-4 (IL-4) recombinant protein, and 500 × protein transport inhibitor cocktail were purchased from Thermo Fisher Scientific Inc. (Massachusetts, USA). OVA_257−264_ peptide was purchased from Shanghai Sangon Biotech Co., Ltd. (Shanghai, P. R. China). Alanine aminotransferase (ALT), aspartate aminotransferase (AST), blood urea nitrogen (BUN), and creatinine (CRE) assay kits were purchased from Nanjing Jiancheng Bioengineering Institute (Jiangsu, P. R. China). Flow cytometry (FCM) antibodies and enzyme-linked immunosorbent assay (ELISA) kits are listed in Table S1.

*2. Cells and animals*

DC2.4 cells (immortalized murine dendritic cells) were cultured in RPMI 1640 medium supplemented with 10% (*V*/*V*) FBS and 1% (*V*/*V*) P/S. Bone marrow-derived dendritic cells (BMDCs) were generated from 6−8-week-old C57BL/6N mice by culturing bone marrow cells for seven days in RPMI 1640 medium supplemented with 10% (*V*/*V*) FBS, 1% (*V*/*V*) P/S, 20.0 ng mL^−1^ GM-CSF, and 10.0 ng mL^−1^ IL-4. LLC-OVA cells (purchased from Vigen Biotechnology, Zhejiang, P. R. China) were cultured in DMEM supplemented with 10% (*V*/*V*) FBS, 1% (*V*/*V*) P/S, and 0.3 μg mL^−1^ puromycin. CT26 (mouse colorectal carcinoma) cells were cultured in DMEM supplemented with 10% (*V*/*V*) FBS and 1% (*V*/*V*) P/S. All cells were maintained at 37 °C in a humidified atmosphere containing 5% (*V*/*V*) carbon dioxide (CO_2_).

Female C57BL/6N mice (6−8 weeks old) were purchased from Beijing Vital River Laboratory Animal Technology Co., Ltd. (Beijing, P. R. China). Female BALB/c mice (6−8 weeks old) were purchased from Changchun Yisi Experimental Animals Technology Co., Ltd. (Changchun, P. R. China). All animal experiments were conducted in accordance with institutional guidelines and were approved by the Animal Welfare and Ethics Committee of the Changchun Institute of Applied Chemistry, Chinese Academy of Sciences.

*3. Characterizations*

Proton (^1^H) and carbon-13 (^13^C) nuclear magnetic resonance (NMR) spectra were recorded using a Bruker Avance 500 MHz spectrometer (Bruker BioSpin GmbH, Rheinstetten, Germany). Fourier transform-infrared (FT-IR) spectra were obtained using a Bio-Rad FTS-600 spectrometer (Bio-Rad, California, USA). Hydrodynamic diameter (*D*_h_) and zeta potential were measured using a Malvern Zetasizer instrument (Malvern Instruments Ltd., Malvern, UK). Morphological features were examined using a JEM-1400 transmission electron microscope (TEM, JEOL Ltd., Tokyo, Japan). Young's moduli were measured using a SCANASYST-FLUID+ atomic force microscope (AFM) (Bruker, California, USA). Fluorescence signals were recorded using a BD flow cytometer (BD Biosciences, California, USA) or an FV4000 confocal laser scanning microscope (CLSM) (Olympus Corporation, Tokyo, Japan).

*4. Synthesis of Cbz-D-Lys NCA*

Cbz-D-Lys (20.37 g) was added to a three-necked flask, which was dried under vacuum for 1 h. Anhydrous THF (320.0 mL) was added to the flask to form a solution. The Cbz-D-Lys solution was then reacted with triphosgene (16.9 g) at 55 °C under a nitrogen (N_2_) atmosphere for 2 h. Next, the reaction solution was blown to 100.0 mL under high-flux N_2_. The reaction solution was then precipitated with an excess of low-temperature *n*-hexane (1,000.0 mL) and filtered through a G4 sand core funnel to obtain the crude product. The crude product was dissolved in low-temperature ethyl acetate (500.0 mL) and transferred to a separatory funnel. The product solution was then washed with a low-temperature saturated sodium chloride solution and pure water to remove insoluble impurities and by-products. The organic phase was transferred to a conical flask, dried over anhydrous magnesium sulfate, and stored overnight at −20 °C. The next day, the anhydrous magnesium sulfate was filtered off, and the filtrate was vacuum-evaporated to yield a white solid product. The final product was recrystallized three times with THF and *n*-hexane to give a white crystalline solid (19.1 g, 85.79%).

*5. Synthesis of L-Phe NCA*

L-Phe NCA was synthesized in the same way as Cbz-D-Lys NCA. L-Phe (31.46g) reacted with triphosgene (46.2 g) in THF (550.0 mL). The final product was a white crystalline solid (29.5 g, 81.03%).

*6. Synthesis of F-_D_K*

L-Phe NCA (0.66, 1.30, and 1.95 g) was added to different Schlenk flasks, dried under vacuum for 1 h, and dissolved in anhydrous DMF (16.0, 23.0, and 31.0 mL). Then, *n*-HA (0.046 g) was added, and the mixture was stirred at room temperature for three days in each Schlenk flask. Next, Cbz-D-Lys NCA (0.78 g) was added to each Schlenk flask, and the reaction was continued for another three days. During this period, gas release was carried out intermittently under waterless conditions. The reaction was then quenched with cold anhydrous ether, centrifuged, and dried.

Subsequently, the above product (F-Cbz-_D_K) (1.0 g) was weighed into a clean flask. F-Cbz-_D_K was stirred with TFA (10.0 mL) and HBr/HAc (3.0 mL) for 1 h to dissolve. After the reaction, the reaction solution was settled with anhydrous ether at a low temperature. The product was dissolved in 30.0 mL of DMF, dialyzed, and lyophilized to obtain the final product (F-_D_K).

*7. Determination of antigen loading efficiencies of F-_D_K*

After F-_D_K complexed OVA, the vaccines were ultracentrifuged at 17,000 *g* for 40 min. The supernatant was collected and analyzed using a BCA kit at 562 nm to determine the amount of unbound OVA. The antigen-loading efficiency (ALE) was calculated using the following equation (Eq. S1).

$$\begin{aligned} \text{ALE }\left( \text{\%} \right)\text{=}\frac{\text{Total antigen }-\text{ Unbound antigen}}{\text{Total antigen}}\text{ × 100\%}\#S\text{1} \end{aligned}$$

*8. In vitro cytotoxicity assay*

DC2.4 cells were seeded at 1.0 × 10^4^ cells per well in 96-well plates. After attachment, the cells were treated with RPMI 1640 medium and supplemented with the indicated F-_D_K or F-_D_K/OVA at concentrations of 0, 0.156, 0.313, 0.625, 1.250, 2.500, 5.000, 10.000, and 20.000 μg mL⁻¹. The plates were incubated for 12 h at 37 °C under 5% (*V*/*V*) CO_2_. Subsequently, the medium was removed, and 100.0 μL of fresh RPMI 1640 containing 10.0 μL of CCK-8 was added. After incubation at 37 °C for 2 to 4 h, the absorbance at 450 nm was measured.

*9. Cellular uptake assay*

DC2.4 cells were seeded at a density of 8.0 × 10^4^ cells per well into 24-well plates. After cell adhesion, the original medium was removed and replaced with culture medium containing OVA-FITC (0.8 μg mL^−1^) or F-_D_K/OVA-FITC (4.0 μg mL^−1^). Incubation was continued for 0, 3, 6, 12, and 24 h, after which the cells were collected for FCM analysis. The FITC fluorescence intensity for each group was analyzed using FlowJo v10.8.1.

For CLSM imaging, DC2.4 cells were seeded at a density of 2.0 × 10^5^ cells per dish into glass-bottom confocal dishes. Following cell adhesion, the original medium was removed and replaced with culture medium containing OVA-Cy5 (0.8 μg mL^−1^) or F-_D_K/OVA-Cy5 (4.0 μg mL^−1^). After culturing for 0, 3, 6, 12, and 24 h, cells were washed twice with 1× PBS. Subsequently, cells were fixed with 4% (*W*/*V*) PFA for 10 min, stained with DiO for 5 min to label the plasma membrane, and counterstained with Hoechst 33342 for 10 min to visualize the nucleus. Finally, cells were maintained in 1× PBS and imaged using standardized confocal parameters through a CLSM.

*10. Lysosomal escape assay*

DC2.4 cells were seeded at a density of 2.0 × 10^5^ cells per dish into glass-bottom confocal dishes. After adhesion, the culture medium was replaced with fresh medium containing OVA-Cy5 (0.8 μg mL^−1^) or F-_D_K/OVA-Cy5 (4.0 μg mL^−1^). Cells were cultured for 8 and 12 h and washed twice with 1× PBS. Cells were then incubated with LysoTracker Green for 45 min at 37 °C to label lysosomes. The nuclei were counterstained with Hoechst 33342 for 10 min at room temperature (RT). After two additional 1× PBS washes, cells were maintained in RPMI 1640 medium and immediately imaged by CLSM to evaluate the colocalization of the vaccines with lysosomes.

*11. RNA sequencing analysis*

Immature BMDCs were seeded at 1.0 × 10^6^ cells per well into 12-well plates and treated with RPMI 1640 medium containing F_15_-_D_K_10_/OVA (8.0 μg mL^−1^) or an equivalent volume of 1× PBS with four replicate wells per group. After 12 h of incubation, BMDCs were gently detached by pipetting, transferred into 15.0 mL centrifuge tubes, and washed twice with 1× PBS. Cells were resuspended in 200.0 μL TRIzol and subjected to repetitive pipetting until complete lysis was achieved. Lysates were transferred to cryovials and stored at −80 °C. Finally, samples were shipped on dry ice to Shanghai Baiqu Biomedical Technology Co., Ltd. for RNA sequencing.

*12. Western blotting analysis*

DC2.4 cells were seeded at 1.5 × 10^6^ cells per well into 6-well plates and treated with RPMI 1640 medium containing OVA (1.6 μg mL^−1^), F_5_-_D_K_10_/OVA (8.0 μg mL^−1^), F_10_-_D_K_10_/OVA (8.0 μg mL^−1^), F_15_-_D_K_10_/OVA (8.0 μg mL^−1^), or an equivalent volume of 1× PBS. Plates were transferred to ice for subsequent steps. Cells were washed twice with ice-cold 1× PBS, residual liquid was removed, and 200.0 μL RIPA containing PMSF, PI, and cocktail (100:1:1:2) was added for complete lysis. Lysates were transferred to 1.5 mL microtubes, centrifuged at 12,000 rpm for 15 min at 4 °C, and the supernatants were collected as total protein extracts. Protein concentration was determined with a BCA kit. Samples were adjusted to equal concentration and volume with RIPA and 5× loading buffer. TLR4 samples were denatured at 37 °C for 45 min, whereas MyD88, NF-κB p65, and NF-κB pp65 samples were denatured at 95 °C for 10 min. Aliquots were stored at −80 °C. Proteins were separated on SDS−PAGE gels at 80 V until the prestained protein marker resolved, then the voltage was increased to 120 V until the dye front neared the gel end. Proteins were transferred to PVDF membranes pre-activated with methanol at 400 mA for 90 min in a 4 °C. Membranes were blocked with protein-free rapid blocking solution for 60 min, washed with Tris-buffered saline containing 0.1% (*V*/*V*) Tween 20 (1× TBST), and incubated overnight at 4 °C with rabbit polyclonal antibody to TLR4 (1:1000), MyD88 (1:1000), NF-κB p65 (1:1000), or NF-κB pp65 (1:1000) diluted in primary antibody diluent buffer. Primary antibodies were recovered, and membranes were washed three times with 1× TBST, then incubated with recombinant rabbit anti-β-actin antibody (1:1000) in the same diluent for 3 h at RT. After antibody recovery, membranes were incubated for 1 h at RT with HRP-conjugated goat anti-rabbit IgG (H+L) (1:5000) diluted in secondary antibody diluent buffer. Following three 1× TBST washes, excess liquid was blotted with filter paper. Membranes were evenly soaked with ECL reagent and imaged using an AI600 imaging system.

*13. Draining lymph nodes migration* in vivo

At −24, −12, and −6 h, separate groups of C57BL/6N mice received a subcutaneous injection of 40.0 μL of Cy7-labeled vaccine formulations F_5_-_D_K_10_/OVA-Cy7, F_10_-_D_K_10_/OVA-Cy7, or F_15_-_D_K_10_/OVA-Cy7 at 500.0 μg mL^−1^ into the right footpad. At 0 h, the DLNs were excised, and the LNs from untreated mice served as controls. Fluorescence intensity was measured and analyzed with an *in vivo* imaging system. Subsequently, one lymph node per group was frozen-sectioned at 10.0 μm, stained with FITC-conjugated anti-CD11c antibody (Servicebio), and imaged by CLSM. To evaluate vaccine uptake by distinct immune subsets in the DLNs, C57BL/6N mice were injected with F-_D_K/OVA-Cy5 using the same method. After 24 h, the DLNs were harvested and mechanically disrupted to generate single-cell suspensions. Cells were stained with Zombie Aqua and fluorescently labeled anti-CD11c, anti-CD3, anti-CD19, and anti-CD11b antibodies and analyzed by FCM using FlowJo v10.8.1.

*14. Biodistribution analysis of F_15_-_D_K_10_/OVA*

C57BL/6N mice received a subcutaneous injection of 200.0 μL of Cy5-labeled vaccine formulation F_15_-_D_K_10_/OVA-Cy5 at 500.0 μg mL^−1^ into the dorsal flank. After 24 h, the injection-site skin, DLN, heart, liver, spleen, lung, and kidney were collected with transcardial perfusion. Tissues were rinsed with PBS, blotted dry, and weighed. For fluorescence quantification, each tissue was homogenized in lysis buffer (0.1 g mL^−1^ tissue for solid organs, 0.01 g mL^−1^ for the DLN, and 0.05 g mL^−1^ for injection-site skin) at 37 °C for 1 h. The tissue homogenate fluorescence was read. A matrix-matched calibration curve was prepared by blank homogenate from mice with known amounts of the same F_15_-_D_K_10_/OVA-Cy5. Unlabeled tissue from mice as background was subtracted. The fluorescence-equivalent percent injected dose per gram of tissue (%ID/g) was calculated using Eq. S2. *M*_Tissue_ is the vaccine formulation dose in the tissue. *M*_Injection_ is the actual delivered dose per mouse, and *m*_Tissue_ is the weight of the tissue.

$$\begin{aligned} \text{\%ID/g = }\frac{\text{M}\text{Tissue}\text{ }}{\text{M}\text{Injection}} \text{× }\frac{\text{1}}{\text{m}\text{Tissue}} \text{× 100\%}\#S\text{2} \end{aligned}$$

*15.* *Cancer therapy assay in CT26 model*

On day 0, female BALB/c mice were subcutaneously injected in the right dorsal flank with 3.0 × 10^5^ CT26 cells. After tumor inoculation, mice were randomly assigned to experimental groups to ensure uniform baseline conditions. On days 2, 5, 9, and 12, mice received subcutaneous injections of 200.0 μL of 1× PBS, CT26-WCL, Alum/CT26-WCL, or F15-DK10/CT26-WCL, each containing 50.0 μg of CT26-WCL. Body weight, tumor length, and width were measured in a blinded manner to avoid subjective bias. These data were recorded at the indicated time points. Tumor volumes were calculated using Eq. S3, and tumor inhibition rates were subsequently determined using Eq. S4. Mice were humanely euthanized when the tumor volume reached 2,000 mm^3^, when severe weight loss (>20%) occurred, or when mice showed obvious mental depression and mobility impairment. No animals or data were excluded from this study, and all collected data were included in the final statistical analysis.

$$\begin{aligned} \text{Tumor volume }\left( \text{mm}\text{3} \right)\text{ = }\frac{\text{Length × Width}\text{2}}{\text{2}}\#S\text{3} \end{aligned}$$

$$\begin{aligned} \text{Tumor inhibition rate}\text{ }\left( \text{\%} \right)\text{ = }\left( \text{1 }-\text{ }\frac{\text{V}\text{Treatment}}{\text{V}\text{Control}} \right)\text{ × 100\%}\#S4 \end{aligned}$$

*16. Complement-dependent cytotoxicity test*

LLC-OVA cells were seeded at 8.0 × 10^3^ cells per well into a 96-well plate overnight. Serum samples diluted 1:64 in culture medium were added to each well and incubated for 2 h at 37 °C. Then, the supernatant was discarded, and the culture media containing 10% (*V*/*V*) rabbit serum complement were added and incubated for 4 h at 37 °C. The supernatant was discarded, and culture medium containing 10% (*V*/*V*) CCK-8 was added. After incubating for 1.5 h at 37 °C, the OD_450_ of each well was read with a microplate reader.

*17. Statistical analysis*

Statistical analysis was performed using GraphPad Prism software (v9.5.0.730). One-way analysis of variance, followed by Tukey's multiple-comparisons test, was used for group comparisons. Survival data were analyzed using Kaplan-Meier curves and the log-rank (Mantel-Cox) test. Data are presented as mean ± standard deviation (SD) unless otherwise specified. A *P* value below 0.05 was considered statistically significant (NS, not significant; **P* < 0.05, ***P* < 0.01, ****P* < 0.001, *****P* < 0.0001).

**Table S1**

Flow cytometry antibodies and ELISA kits information.

| Product | Manufacture | Catalog number |
| --- | --- | --- |
| Zombie Aqua^TM^ Fixable Viability Kit | Biolegend | 423102 |
| CD11c Monoclonal Antibody, FITC | eBioscience | 11-0114-82 |
| CD11c Monoclonal Antibody, APC/Cyanine 7 | Biolegend | 117324 |
| CD80 Monoclonal Antibody, Brilliant Violet 650 | Biolegend | 104732 |
| CD40 Monoclonal Antibody, Brilliant Violet 421 | Biolegend | 124641 |
| MHC Class II (I-A/I-E) Monoclonal Antibody, PE/Cyanine 7 | Biolegend | 107630 |
| MHC Class II (I-A/I-E) Monoclonal Antibody, APC | Biolegend | 107614 |
| SIINFEKL peptide bound to H-2Kb Monoclonal Antibody, PE | eBioscience | 12-5743-82 |
| CD19 Monoclonal Antibody, Brilliant Violet 650 | Biolegend | 115541 |
| CD11b Monoclonal Antibody, APC | eBioscience | 17-0112-82 |
| CD86 Monoclonal Antibody, PE | Biolegend | 105007 |
| CD86 Monoclonal Antibody, eFluor450 | eBioscience | 48-0862-82 |
| CD3a Monoclonal Antibody, FITC | Biolegend | 100204 |
| CD4 Monoclonal Antibody, Brilliant Violet 421 | Biolegend | 100543 |
| CD4 Monoclonal Antibody, Super Bright 600 | eBioscience | 63-0041-82 |
| CD8a Monoclonal Antibody, PE/Cyanine 7 | eBioscience | 25-0081-82 |
| IFN-γ Monoclonal Antibody, APC | eBioscience | 17-7311-82 |
| IFN-γ Monoclonal Antibody, PE | eBioscience | 12-7311-82 |
| IL-4 Monoclonal Antibody, PE | Biolegend | 504103 |
| CD69 Monoclonal Antibody, APC | Biolegend | 104514 |
| CD44 Monoclonal Antibody, PE | eBioscience | 12-0441-82 |
| CD62L Monoclonal Antibody, APC | eBioscience | 17-0621-82 |
| TNF-α Monoclonal Antibody, APC | eBioscience | 17-7321-82 |
| CD45 Monoclonal Antibody, APC/Cyanine 7 | eBioscience | A15395 |
| Foxp3 Monoclonal Antibody, PE | eBioscience | 12-5773-82 |
| CD25 Monoclonal Antibody, APC | eBioscience | 17-0251-82 |
| F4/80 Monoclonal Antibody, PE/Cyanine 7 | eBioscience | 25-4801-82 |
| F4/80 Monoclonal Antibody, PE | Biolegend | 123110 |
| CD206 Monoclonal Antibody, FITC | Biolegend | 141703 |
| NK1.1 Monoclonal Antibody, PE | Biolegend | 108707 |
| Ly-6G/Ly-6C (Gr-1) Monoclonal Antibody, PerCP/Cyanine 5.5 | Biolegend | 108427 |
| Mouse IFN-γ Uncoated ELISA Kit | Invitrogen | 88-7314-88 |
| Mouse IL-12p70 Uncoated ELISA Kit | Invitrogen | 88-7121-88 |
| Mouse IL-6 Uncoated ELISA Kit | Invitrogen | 88-7064-88 |
| Mouse TNF-α Uncoated ELISA Kit | Invitrogen | 88-7324-88 |
| Mouse Granzyme B ELISA Kit | Tianjin Anoric | 318230215 |
| Mouse Perforin ELISA Kit | Tianjin Anoric | 652230215 |
| Mouse IL-2 Uncoated ELISA Kit | Jonln | JL20256 |

**Table S2.** GPC results of F_5_-Cbz-_D_K_10_, F_10_-Cbz-_D_K_10_, and F_15_-Cbz-_D_K_10_.

| Polymers | *M*_n_ (g mol^−1^) | *M*_w_ (g mol^−1^) | PDI |
| --- | --- | --- | --- |
| F_5_-Cbz-_D_K_10_ | 7300 | 7700 | 1.06 |
| F_10_-Cbz-_D_K_10_ | 9300 | 9800 | 1.05 |
| F_15_-Cbz-_D_K_10_ | 14000 | 14500 | 1.04 |

**Fig. S1.** Characterizations of L-Phe NCA. (A) Synthetic routes, (B) ^1^H NMR spectra, and (C) ^13^C NMR spectra of L-Phe NCA in CDCl_3_.

**Fig. S2.** Characterizations of Cbz-D-Lys NCA. (A) Synthetic routes, (B) ^1^H NMR spectra, and (C) ^13^C NMR spectra of Cbz-D-Lys NCA in CDCl_3_.

**Fig. S3.** Chemical structural formula, ^1^H NMR spectra, and spectra annotation of F_5_-Cbz-_D_K_10_, F_10_-Cbz-_D_K_10_, and F_15_-Cbz-_D_K_10_ in TFA-*d*.

**Fig. S4.** FT-IR spectra of F_5_-Cbz-_D_K_10_, F_10_-Cbz-_D_K_10_, and F_15_-Cbz-_D_K_10_.

**Fig. S5.** DLS and TEM images of F_5_-_D_K_10_, F_10_-_D_K_10_, and F_15_-_D_K_10_ nanoparticles.

**Fig. S6.** OVA loading efficiencies of F_5_-_D_K_10_, F_10_-_D_K_10_, and F_15_-_D_K_10_ at a concentration ratio of 5:1 (F-_D_K: OVA). Data are represented as mean ± SD (*n* = 5; NS, not significant).

**Fig. S7.** Stability of F-_D_K/OVA nanoparticles. DLS of F-_D_K/OVA nanoparticles under different conditions: pH 7.4, no FBS; pH 5.5, no FBS; and pH 7.4, 50% (*V*/*V*) FBS at 0, 6, and 24 h.

**Fig. S8.** Zeta potential of OVA, F_5_-_D_K_10_, F_10_-_D_K_10_, F_15_-_D_K_10_, F_5_-_D_K_10_/OVA, F_10_-_D_K_10_/OVA, and F_15_-_D_K_10_/OVA. Data are represented as mean ± SD (*n*=3; NS, not significant; **P* < 0.05, ***P* < 0.01, ****P* < 0.001).

**Fig. S9.** Results of cytotoxicity test. (A) Relative cell viability of DC2.4 cells was evaluated 12 h post-treatment with different concentrations of F_5_-_D_K_10_, F_10_-_D_K_10_, and F_15_-_D_K_10_. (B) Relative cell viability of BMDCs was evaluated 24 h post-treatment with F_5_-_D_K_10_/OVA, F_10_-_D_K_10_/OVA, or F_15_-_D_K_10_/OVA (8.0 μg mL^−1^). Data are represented as mean ± SD (*n* = 6 for A, *n* = 3 for B).

**Fig. S10.** DC2.4 cell endocytosis following treatment with fluorescence-labeled vaccines. (A) FACS gating strategy for endocytosis. (B) Counts of FITC^+^ DC2.4 cells and (C) quantitative assessment of mean fluorescence intensity in DC2.4 cell endocytosis following treatment with FITC-labeled vaccines at 0, 1, 3, 6, 12, and 24 h. Quantitative analysis of (D) endocytosis and (E) lysosome escape by CLSM images. The statistical data are represented as mean ± SD (*n* = 4−7 cells for D; *n* = 2−4 cells for E).

**Fig. S11.** FACS analysis of BMDC maturation *in vitro*. (A) FACS gating strategy for BMDC maturation *in vitro*. (B) CD11c^+^CD40^+^ cells following incubation with different treatments for 12 h.

**Fig. S12.** DC activation with TLR inhibitors. (A) CD11c^+^CD80^+^ of DC2.4 cells pretreated with TAK-242 (5.0 μM) or without TAK-242 for 2 h and then incubated with PBS, LPS, or F-_D_K for 12 h. (B) Concentrations of TNF-α in supernatant of BMDC pretreated with C29 (150.0 μM) and HCQ (10.0 μM) for 1 h, then incubated with F_15_-_D_K_10_ for 12 h. Data are represented as mean ± SD (*n* = 3; NS, not significant; ***P* < 0.01, *****P* < 0.0001).

**Fig. S13.** Western blot analysis of TLR4, MyD88, NF-κB p65, NF-κB pp65, and β-actin proteins in DC2.4 cells with different treatments for 12 h.

**Fig. S14.** Semi-quantitative analysis of different immune cellular uptake in DLNs with different treatments at 24 h. Data are represented as mean ± SD (*n* = 3; NS, not significant; **P* < 0.05).

**Fig. S15.** *In vivo* distribution of F-_D_K/OVA. (A) Fluorescence imaging and (B) semi-quantitative analysis of the heart, liver, spleen, lung, kidneys, and inguinal LNs after subcutaneous injection of F_15_-_D_K_10_/OVA-Cy5 at 12, 24, and 48 h. (C) Biodistribution of F_15_-_D_K_10_/OVA-Cy5 in heart, liver, spleen, lung, kidney, and inguinal DLN at 24 h post-subcutaneous administration. Data are represented as mean ± SD (*n* = 3 for A−C; NS, not significant; **P* < 0.05, ****P* < 0.001).

**Fig. S16.** FACS gating strategy for analysis of DC antigen cross-presentation in DLNs.

**Fig. S17.** FACS analysis of T cells in DLNs. (A) FACS gating strategy for Th1 and Th2 cells in CD4^+^ T cells and IFN-γ^+^CD8^+^ T cells. (B) IFN-γ^+^CD8^+^ T cells in DLNs on day 5 after different treatments.

**Fig. S18.** FACS analysis of B cell and T cell maturation in DLNs. (A) FACS gating strategy for B cell and T cell maturation. (B) CD86^+^ B cells in DLNs on day 5 after different treatments.

**Fig. S19.** Individual tumor growth curves. Tumor volumes of mice injected with (A) PBS, (B) OVA, (C) F_5_-_D_K_10_/OVA, (D) F_10_-_D_K_10_/OVA, and (E) F_15_-_D_K_10_/OVA from day 0 to 31 in the LLC-OVA therapeutic model. (*n* = 7 for A−E).

**Fig. S20.** Weights of tumors with different treatments on day 31. Data are represented as mean ± SD (*n* = 5; NS, not significant; **P* < 0.05, ****P* < 0.001).

**Fig. S21.** FACS gating strategy for analysis of CD4^+^ T cells, CD8^+^ T cells, and Treg cells in tumors.

**Fig. S22.** FACS gating strategy for analysis of TAM, M1, and M2 cells in tumors.

**Fig. S23.** FACS gating strategy for analysis of MDSCs and NK cells in tumors.

**Fig. S24.** Serum biochemical indicators of F-_D_K/OVA. (A) Levels of ALT, (B) AST, (C) BUN, (D) CRE, (E) TNF-α, and (F) IFN-γ in the serum of mice with different treatments. Data are represented as mean ± SD (*n* = 5; NS, not significant; **P* < 0.05, ***P* < 0.01, ****P* < 0.001).

**Fig. S25.** H&E analysis of the heart from the PBS, OVA, F_5_-_D_K_10_/OVA, F_10_-_D_K_10_/OVA, and F_15_-_D_K_10_/OVA groups.

**Fig. S26.** H&E analysis of the liver from PBS, OVA, F_5_-_D_K_10_/OVA, F_10_-_D_K_10_/OVA, and F_15_-_D_K_10_/OVA groups.

**Fig. S27.** H&E analysis of the spleen from PBS, OVA, F_5_-_D_K_10_/OVA, F_10_-_D_K_10_/OVA, and F_15_-_D_K_10_/OVA groups.

**Fig. S28.** H&E analysis of the lung from PBS, OVA, F_5_-_D_K_10_/OVA, F_10_-_D_K_10_/OVA, and F_15_-_D_K_10_/OVA groups.

**Fig. S29.** H&E analysis of the kidney from PBS, OVA, F_5_-_D_K_10_/OVA, F_10_-_D_K_10_/OVA, and F_15_-_D_K_10_/OVA groups.

**Fig. S30.** FACS gating strategy for analysis of T_CM_ cells in the spleens.

**Fig. S31.** FACS gating strategy for analysis of IFN-γ^+^ and TNF-α^+^ of CD8^+^ T cells in the spleens in splenocyte restimulation assays.

**Fig. S32.** Immunological effects of F-_D_K/OVA. (A) CD40^+^ of B cells in the DLNs, (B) CD40^+^ of B cells, and (C) CD86^+^ of B cells in the spleen after two different treatments. Data are represented as mean ± SD (*n* = 5 for A−C; NS, not significant; **P* < 0.05, ***P* < 0.01, *****P* < 0.0001).

**Fig. S33.** Individual tumor growth curves. Tumor volumes of mice injected with (A) PBS, (B) OVA, (C) F_5_-_D_K_10_/OVA, (D) F_10_-_D_K_10_/OVA, or (E) F_15_-_D_K_10_/OVA from day 0 to 322 in the LLC-OVA prevention model. (*n* = 12).

**Fig. S34.** Photograph of faint scars at tumor-growing site in tumor-free mice injected with F-_D_K/OVA after the third tumor challenge.

**Fig. S35.** Biosecurity of F-_D_K/OVA. Weights of mice receiving various vaccines from (A) days 121−191 and (B) days 287−347.

**Fig. S36.** H&E analysis of the heart, liver, spleen, lung, and kidney from F_15_-_D_K_10_/OVA group on day 644.
